# Transcriptomics and Proteomics Reveal That TLPW Acupuncture Ameliorates Proteinuria in Diabetic Kidney Disease Model Rats by Suppressing Epithelial-to-Mesenchymal Transition via the DPP4/SDF-1*α*/TGF-*β*/Smad Signalling Axis

**DOI:** 10.1155/jdr/2379872

**Published:** 2025-10-01

**Authors:** Yue Ji, Jiya Sun, Zihao Zhuang, Yunming Xiao, Shipian Li, Zhilong Zhang, Xu Wang, Xinju Li

**Affiliations:** ^1^Key Laboratory of Dongzhimen Hospital, Beijing University of Traditional Chinese Medicine, Beijing, China; ^2^College of Traditional Chinese Medicine, Tianjin University of Traditional Chinese Medicine, Tianjin, China; ^3^Department of Acupuncture and Moxibustion, Shanghai Jiading Hospital of Traditional Chinese Medicine, Shanghai, China; ^4^Department of Nephrology, Medical School of Chinese PLA, First Medical Center of Chinese PLA General Hospital, Beijing, China; ^5^Department of Acupuncture and Moxibustion, Tianjin Academy of Traditional Chinese Medicine Affiliated Hospital, Tianjin, China

## Abstract

Ten male Sprague–Dawley rats were randomly assigned to serve as the negative control (NC) group, and 50 others were maintained on a high-fat diet (HFD). After 18 weeks of feeding, the HFD group received streptozotocin (STZ) intraperitoneally to establish a DKD model. HFD group rats (24-h urinary protein excretion rate ≥ 30 mg) were randomly divided into model (DKD), tiaolipiwei acupuncture (DKD + Acu, acupuncture for 30 min), AMD3100 (DKD + AMD3100, AMD3100 intraperitoneal injection) and tiaolipiwei acupuncture + AMD3100 group (DKD + Acu + AMD3100, AMD3100 intraperitoneal injection combined with tiaolipiwei acupuncture for 30 min) groups. The intervention lasted 4 weeks, and body weight and random blood glucose levels were recorded for each group before treatment each week. Postintervention (at 4 weeks), urine was collected to assess urinary protein–creatinine ratios, 24-h urinary protein contents and urinary podocyte injury–related enzyme levels. Renal cortex tissues from three to five rats in the control, DKD and DKD + Acu groups were sent for transcriptomic and proteomic analyses, and renal tissues were collected for analyses of pathological indicators and mechanisms. Twenty-four-hour Upro, 24-h urinary Spondin 2 (SPON2) levels, UACRs and random serum creatinine, urea nitrogen and blood glucose levels in the DKD + Acu group exhibited significantly reduced levels compared to the DKD group (#*p* < 0.05). According to the transcriptomic 2.2.2s and proteomic results, immunofluorescence or western blotting was used to assess podocyte-specific marker expression levels (nephrin, podocin and CD2AP), epithelial–mesenchymal transition (EMT) markers (desmin, Fsp1 and *α*-SMA) and DPP-4/SDF-1*α*/TGF-*β*/Smad signalling axis components; nephrin, podocin and CD2AP expression significantly elevated (#*p* < 0.05 or ##*p* < 0.01) and desmin, Fsp1 and *α*-SMA expression greatly decreased (#*p* < 0.05 or ##*p* < 0.01) in the DKD + Acu group. Tiaolipiwei acupuncture regulated the Dpp-4/SDF-1*α*/TGF-*β*/Smad signalling axis (#*p* < 0.05 or ##*p* < 0.01), but this effect was reduced by AMD3100 (@*p* < 0.05 or @@*p* < 0.01). Tiaolipiwei acupuncture modulates the DPP-4/SDF-1*α*/TGF-*β*/Smad signalling axis to inhibit podocyte EMT and alleviate podocyte and renal injury, ultimately ameliorating proteinuria in DKD model rats.

## 1. Introduction

Diabetic kidney disease (DKD) describes a kidney disease with specific pathological structural and functional changes caused by chronic hyperglycaemia. DKD represents a major microvascular complication of diabetes mellitus and a leading cause of end-stage renal disease (ESRD) globally [1]. According to statistics from the International Diabetes Association, global diabetes prevalence affected an estimated 537 million individuals in 2021, with projections indicating a surge to 643 million cases by 2030 [2]. Without intervention, diabetes will greatly increase the risk of premature death caused by ESRD and is related to an increase in cardiovascular mortality [3]. Therefore, the effective prevention and treatment of DKD have important scientific value and social significance.

DKD is a complex and heterogeneous disease, the core histopathological characteristics of which are mesangial cell hypertrophy, glomerular extracellular matrix deposition and podocyte loss [4], and the pathogenesis is mainly related to renal haemodynamic factors [5], metabolic disorders [6], oxidative stress [7], inflammatory mechanisms [8], genetics and epigenetics [9], autophagy [10], etc. There is also evidence that podocyte loss and redifferentiation and epithelial dysfunction are amongst the factors leading to DKD recurrence [4]. At present, modern medical treatments for DKD mainly concentrate on controlling blood pressure, regulating blood glucose, improving lipid metabolism, inhibiting the RAS and using angiotensin-converting enzyme inhibitor (ACEI)/angiotensin receptor blocker (ARB) drugs such as SGLT-2 inhibitors [11], but the progression of DKD still cannot be effectively controlled.

The advantages of traditional Chinese medicine (TCM) are uniquely evident in improving the clinical symptoms and pathological indicators of DKD patients. As a representative treatment of the TCM tradition, acupuncture is a treasure of the Chinese nation and an important means of nondrug therapy and is also considered a part of complementary alternative medicine by Western countries. Recently, mechanistic studies based on the effect of acupuncture have shown that acupuncture can ameliorate DKD progression by improving the clinical symptoms and haemorheology of DKD patients [12], reducing the inflammatory response [13], promoting renal autophagy [14], reducing blood glucose and lipid levels and inhibiting podocyte activation [15].

However, the generation of acupuncture effects is complex and involves the selection and compatibility of acupoints, complex local dynamic mechanisms of acupoints, multiple links and numerous substances that range from topical to systemic. Based on long-term clinical practice and epidemiological investigation, Professor Zhang Zhilong, the inheritance director of the nationally famous TCM studio, created ‘tiaolipiwei (TLPW) acupuncture' (spleen and stomach-regulating acupuncture method) to prevent and treat DKD by supplementing the spleen and kidney and distinguishing turbidity.

Previously, we conducted clinical trials [16] via multicentre, randomized controlled and blind studies, which indicated that TLPW acupuncture early intervention can not only alleviate the symptoms and signs of diabetic nephropathies but also exert a favourable regulatory impact on lipid metabolism and glucose, along with the estimated glomerular filtration rate (eGFR) and levels of urinary albumin. In addition, our prior fundamental investigation indicates that the application of tiaolipiwei acupuncture can improve podocyte damage, leading to a decrease in albuminuria and hindering the advancement of DN in an animal model [17].

In this study, we utilized a rat model of Type 2 diabetic nephropathy induced by a high-fat diet combined with STZ (HFD/STZ-induced DN). The treatment group received TLPW acupuncture for 4 weeks, and the potential targets were identified by RNA-Seq and TMT quantitative proteomic analysis and verified by molecular biological methods. We speculate that TLPW acupuncture ameliorates proteinuria in DKD rats by suppressing epithelial-to-mesenchymal transition through the DPP4/SDF-1*α*/TGF-*β*/Smad signalling axis, as validated in further animal experiments.

## 2. Materials and Methods

### 2.1. Animals and Reagents

The following animals and reagents were used: Sprague–Dawley (SD) rats (Huafukang, China; SCXK 2019-0009), streptozotocin (STZ, Sigma-Aldrich Chemical Co., United States), sodium citrate buffer salt solution (Beijing Solaibao Technology Co. Ltd), urine protein qualitative kit and urine protein quantification kit (Nanjing Jiancheng Biological Engineering Research Institute Co. Ltd), ACCU-CHEK glucose metre (Roche), AMD3100 (Sigma-Aldrich Chemical Co., United States) and disposable sterile stainless steel needles (0.25 mm ID × 25 mm length, Suzhou Medical Supplies Factory Co. Ltd).

### 2.2. Animals and Treatment

#### 2.2.1. Laboratory Animals and Feeding Environment

Sixty male SD rats weighing 140–160 g were housed in individual cages within a temperature-controlled room (23 ± 2°C) with regulated humidity levels (55 ± 10%). The rats were subjected to a 12-h light (07:00–19:00)/dark cycle. They had unrestricted access to water and food for the duration of the study. The Ethics Committee of Tianjin University of TCM granted approval for the in vivo studies protocol (IRM-DWLL-2019103), which adhered to the NIH publication (eighth edition, updated 2011): Guide for the Care and Use of Laboratory Animals.

#### 2.2.2. Animal Grouping and Modelling

Randomly, all rats were allocated to either the control group (*n* = 10) or the diabetic group (*n* = 50). We utilized a HFD and administered low-dose intraperitoneal STZ as the DKD model. In brief, rats were fed a HFD for an 18-week duration. Subsequently, diabetes was induced in the rats belonging to the diabetic group through STZ (25 mg/kg, dissolved in citrate buffer with pH value of 4.3) which was administered intraperitoneally as a single dose. The experimental group was injected with an isovolumetric amount of vehicle solution. Rats with fasting tail blood glucose concentration exceeding 16.7 mmol/L for 3 consecutive days were confirmed to have diabetes 1 week following administration. We used metabolic cages to collect rat urine 5 weeks after the injection to measure both protein concentration and volume. DKD was defined as having a urinary protein level ≥ 30 mg over a period of 24 h.

Randomly, all DKD rats were divided into DKD, DKD + AMD3100, DKD + Acu and DKD + AMD3100 + Acu groups. Both DKD + Acu + AMD3100 and DKD + Acu groups received TLPW acupuncture treatment qd. Moreover, rats in the DKD + AMD3100 and DKD + Acu + AMD3100 groups received daily intraperitoneal injections of AMD3100 (1 mg/kg) for 4 weeks, whilst the control, DKD and DKD + Acu groups received injections of the same volume of PBS. After 4 weeks of treatment, we collected 24-h urine samples from rats, anaesthetized them with 2%–3% isoflurane and euthanized them. The left kidney and whole body were weighed, and renal tissues and blood were obtained for subsequent analysis.

#### 2.2.3. Acupuncture Procedure

The rats were positioned in a supine posture and securely fastened using a nonobstructive strap to ensure that their natural breathing was not affected. Acupuncture treatment was administered at specific acupoints, including Zhongwan (CV12), Taichong (LR3), Yinlingquan (SP9), Xuehai (SP10), Diji (SP8), Sanyinjiao (SP6), Fenglong (S40), Quchi (LI11, bilateral), Hegu (LI4) and Zusanli (ST36), following the experimental animal acupuncture manual. We inserted the needle perpendicularly to a depth ranging from 3 to 4 mm and maintained it for 30 min. Acupuncture was administered using the twirling technique, with an average speed of 120 rotations per minute, for 30 s. Acupuncture sessions were conducted qd over a period of 4 weeks. Following the treatment, all rats were euthanized immediately.

### 2.3. Indicators and Detection Methods

#### 2.3.1. General Statement

On the 23rd week after the establishment of the HFD/STZ-induced DKD rat model, an equal number of rats (seven) were selected at random from each group for weight measurement, and blood samples were obtained from their tails to conduct biochemical tests on blood glucose levels (ACCU-CHEK, China) once a week until the intervention was finished. Simultaneously, we introduced the rats to a metabolic cage for 24-h urine collection. The collected urine was subjected to biuret reaction on a weekly basis throughout the intervention period to assess urinary protein levels over a 24-h period.

#### 2.3.2. Serum Biochemistry and the Kidney Index

On the 4th week after the intervention, all groups of rats were euthanized using 2% sodium pentobarbital anaesthesia, and serum samples were obtained via the abdominal aortic method to measure levels of total cholesterol (TC), triglyceride (TG), serum creatinine (SCR) and blood urea nitrogen (BUN) using an automated biochemical analyzer. The blood samples were centrifuged (3000 rpm, 15 min, 4°C) to prepare serum, after which the serum was extracted and stored (−80°C). The body weight and kidney weight were recorded. The left kidney index (left kidney weight/body weight, LKW/BW) is expressed as a percentage.

#### 2.3.3. Histological Analysis and Immunofluorescence (IF)

As mentioned above, following the collection of blood and urine samples, we sacrificed all animals by cervical dislocation. We fully exposed the kidneys and carefully separated them from the surrounding tissue and renal capsule. Then, a sharp blade was used to make a longitudinal incision along the kidney tissues, and the tissues were harvested and fixed in 10% formalin for the detection of the pathological changes with haematoxylin and eosin (HE) staining, periodic acid–Schiff (PAS) staining and IF. We then embedded kidney tissues in paraffin and subsequently cut them into 5 *μ*M sections for staining with HE (Beijing Bioss Biotechnology Co. Ltd.) and PAS (Microscopy PAS staining kit, Merck Millipore). The expression of nephrin (1:100), podocin (1:100), CD2AP (1:100), desmin (1:100), Fsp1 (1:100), *α*-SMA (1:100) and TGF-beta 1 (1:100) (antibody information refers to 2.3.7 western blot analysis) was detected via immunohistochemical examination with the streptavidin–peroxidase (SP) method. To assess the presence of podocyte injury in DN, transmission electron microscopy (TEM) was performed using a Hitachi H-7650 system (Hitachi, Japan). In a sterile environment, the capsules were separated from the underlying kidney parenchyma, and the renal cortex was extracted with a sterilized scalpel. From the longitudinal section of the kidney, 1 mm^3^ tissue blocks were cut from the cortical edge with ophthalmic scissors and fixed with glutaraldehyde. Using the Epon 812 protocol (SPI Corporation, United States), samples were fixed, stained, sliced into sections that were 70 nm thick and observed under electron microscopy after processing. The analysis focused on evaluating the podocyte foot processes (FPs), FP width and glomerular basement membrane thickness as potential markers for assessing injury and recovery.

#### 2.3.4. Enzyme-Linked Immunosorbent Assay

We measured the levels of Dpp-4 in serum and kidney tissues. The levels of SPON2 were quantified in the 24-h urine samples. ELISA kits (TSZ, United States, and Wuhan Enzyme Free Biotechnology, China) were measured with a plate reader (Thermo, United States) with an experimental reagent kit and quantified by an enzyme-linked double antibody sandwich technique with a detection limit of 1.25–80 pg/mL, a sensitivity of 0.31 pg/mL, a testing time of 4 h, a detection wavelength of 450 nm and a sample volume of 100 *μ*L.

#### 2.3.5. Transcriptomic Analysis

Total RNA was extracted from rat renal cortex tissue with the TRIzol reagent kit (Invitrogen, Carlsbad, CA, United States). An Agilent 2100 Bioanalyzer (Agilent Technologies, Santa Clara, CA, United States) was employed to ensure quality control. Poly mRNA was isolated from the total RNA with oligo (dT) beads. Fragmented mRNA was converted to cDNA through reverse transcription. Subsequently, the constructed library underwent sequencing on the Illumina HiSeq 2500 platform (Illumina, San Diego, CA, United States), which was performed by Novogene Technology Co. Ltd. (Beijing, China). The quantification of transcript expression was performed with RNA-Seq by expectation–maximization (RSEM) software. Transcripts demonstrating a fold change exceeding 1 and a false discovery rate (FDR) lower than 0.05 were stratified as indicative of differential expression. The differentially expressed genes (DEGs) were subsequently analyzed using Kyoto Encyclopedia of Genes and Genomes (KEGG) pathway enrichment (see Supporting Information).

#### 2.3.6. TMT-Labelled Proteomic Analysis

Renal cortex tissues from three rats in each group were transferred into 4× lysis buffer. Then, lysis was completed via ultrasonication, and proteins were quantified with a BCA Protein Assay Kit. The samples underwent homogenization, followed by centrifugation. They were then treated with TCEP for reduction and iodoacetamide for alkylation. We digested protein solutions with trypsin and labelled them with TMT tags according to the instructions provided by the manufacturer (90110, Thermo Fisher Scientific, Waltham, MA, United States). Peptides were separated at pH 10.0 using a Dionex Ultimate 3000 UPLC system (same as above) and subsequently analyzed on an Easy-nLC 1000, which was interfaced with a Q Exactive Hybrid Quadrupole-Orbitrap mass spectrometer (same as above). After trypsin digestion and TMT/iTRAQ labelling according to the TMT kit/iTRAQ kit protocols, LC-MS/MS analysis was performed according to its specific protocol and parameters on an Easy-nLC 1000 UPLC system. The peptides underwent analysis with an NSI source, followed by tandem mass spectrometry (MS/MS) (Q Exactive Plus, Thermo Fisher), which was connected online to the UPLC system. The electrospray ionization (ESI) source was operated at 2.0 kV and was maintained throughout the experiment. Full-spectrum acquisition was performed across a mass-to-charge ratio window of 350–1800, with undigested peptides analyzed in the Orbitrap mass analyzer under 70,000 resolving power. Precursor ions selected for fragmentation via higher energy collisional dissociation (HCD) were subjected to tandem MS using 28% normalized collision energy. Then, with a resolution of 17,500 data in the Orbitrap depending on the process test, the process involves an MS scan followed by 20 MS/MS scans, with dynamic ruled out for 15.0 s. The AGC was adjusted to a value of 5E4 for automatic gain control. The first mass was fixed at 100 m/z. To analyze the MS/MS data results, we utilized the MaxQuant search engine (V.1.5.2.8). MS/MS spectral data were matched to integrated repositories comprising Gene Ontology (GO), KEGG pathways and reversed sequence decoy entries. Trypsin (protease) served as the proteolytic agent, with a tolerance of ≤ 2 enzymatic cleavage omissions. The initial precursor ion identification employed a 20 ppm mass error window, which was tightened to 5 ppm for refined analysis. Fragment ion matches were accepted within 0.02 Dalton deviations. Permanent carbamidomethylation of cysteine residues was enforced, whilst methionine oxidation was permitted as a dynamic modification. A FDR of less than 1% was applied, whilst peptides with a minimum score greater than 40 were considered. Omics sample sizes were determined by pilot data variability, whilst biochemical assays employed larger cohorts to ensure statistical power for parametric tests. For details, see Supporting Information.

#### 2.3.7. Western Blot Analysis

Approximately 100 mg of renal tissue was disrupted in 1 mL of RIPA lysis buffer (Perseebio, PBW003W0) at a temperature below freezing, along with the addition of 1 mM PMSF (Perseebio, PBW007W0). BCA Assay Kits (Perseebio, PBW011W1) were used to determine the protein concentration. Proteins from renal tissues were separated by 4%–12% precast gel (LABLEAD, P41215, P00815) electrophoresis (Perseebio, PBW030W1) and transferred (Perseebio, PBW031W0) onto polyvinylidene difluoride (PVDF) membranes (Millipore, United States). A blocking solution of TBST and 5% defatted milk (Perseebio, PBW034W1) was applied to PVDF membranes for 1 h under standard laboratory conditions, after which nephrin (1:500, Abcam, ab216341), podocin (1:1200, Abcam, ab50339), CD2AP (1:800, CST, #2135), desmin (1:900, CST, #4024), Fsp1 (1:1200, Abcam, ab220213), *α*-SMA (1:1000, CST, #9513), DPP4 (1:800, Abcam, ab187048), stromal cell–derived factor-1*α* (SDF-1*α*) (1:1500, CST, #3530), TGF-*β*1 (1:1000, Abcam, ab215715), p-Smad3 (1:500, CST, #9520), Smad3 (1:1000, CST, #9513) and GAPDH (1:2000, Utibody, UM4002) antibodies were introduced at 4°C overnight. Subsequently, the cells were washed and incubated for 1 h at room temperature with the corresponding secondary antibodies (bs-0295G-HRP, 1:5000). The ECL Plus special sensitive chemiluminescence reagent (Perseebio, PBW041W0) was employed to detect signals. An automatic imaging system (ProteinSimple, United States) was utilized for image acquisition, whilst ImageJ was used for signal quantification.

#### 2.3.8. Statistical Analysis

We performed the data analysis by GraphPad Prism 8.0 and expressed the data as the mean ± SD. We used one-way ANOVA to analyze the data, and the results were considered statistically significant based on a criterion of *p* < 0.05. We used SPSS (SPSS statistical software, IBM) for data processing and visualization.

## 3. Results

### 3.1. Characteristics of Rats With HFD/STZ-Induced DKD

**Figure 1:**
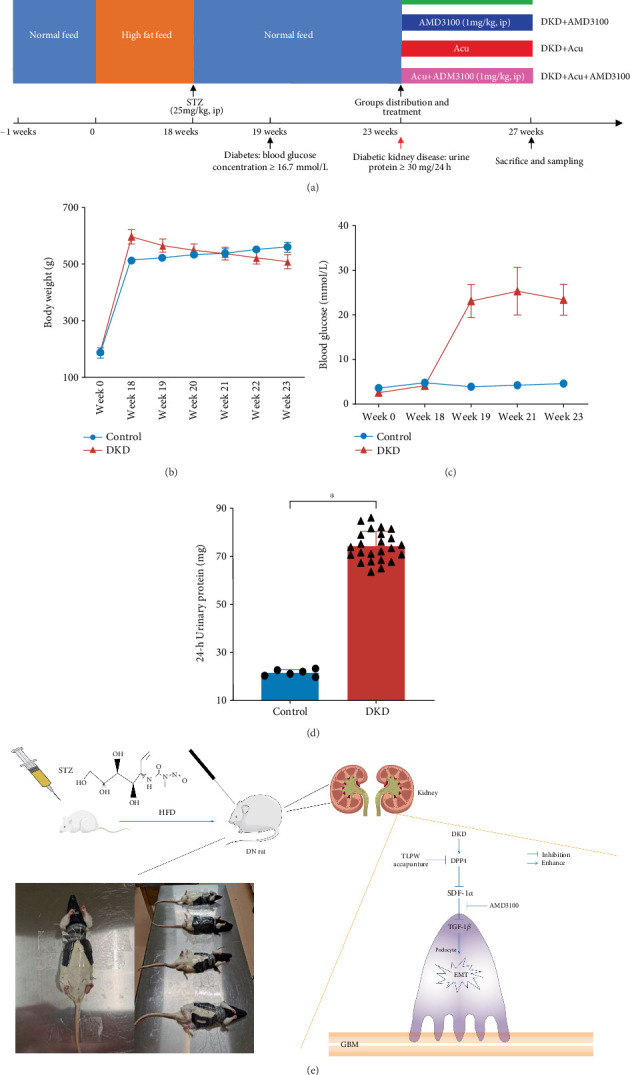
Characteristics of rats with high-fat diet-induced and STZ-induced DKD and the study flow chart. (a) Protocol of experiments involving rats with STZ-induced DKD. (b) Body weight from Week 0 to Week 23 in the control group and DKD group. (c) Blood glucose levels from Week 0 to Week 23 in the control group and DKD group. (d) Twenty-four-hour urinary protein levels in the control group and DKD group. (e) Schematic diagram of the TLPW acupuncture process and study flow chart. ⁣^*^p < 0.05 versus control group.

The study protocol of the experiments in STZ-induced DKD rats is shown in Figure 1a. We established a rat model of HFD and low-dose intraperitoneal STZ-induced DKD. One week after STZ treatment, blood glucose levels > 16.7 mmol/L at 24-h intervals for 3 days were defined as diabetes. Four weeks later, a 24-h urinary protein level higher than 30 mg was described as DKD. Eventually, 36 rats successfully established DKD models and were randomly allocated into four groups (*n* = 9 per group). In the process of DKD modelling, we observed that the 24-h urinary protein concentration, blood glucose level and body weight of the DKD group were significantly changed. At week 23, we detected that the increase in body weight of the DKD group had slowed (Figure 1b). Furthermore, the DKD group exhibited blood glucose levels exceeding 16.7 mmol/L, which are greatly elevated compared with the control group (Figure 1c). Additionally, the urinary protein concentration in DKD rats increased and surpassed 30 mg within a 24-h period (Figure 1d).

### 3.2. TLPW Acupuncture Ameliorated Proteinuria and Improved Lipid Metabolism and Glucose in DKD Rats

**Figure 2:**
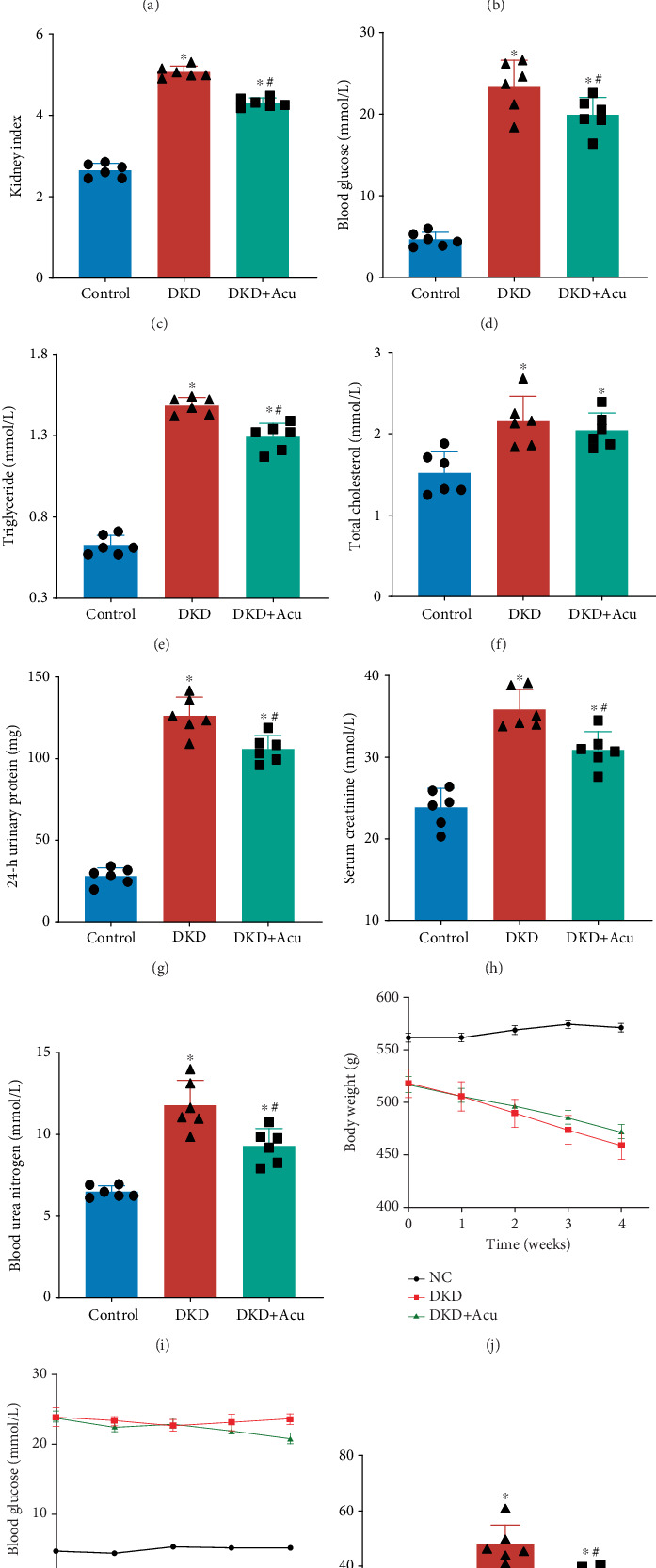
General conditions and biochemical indices at 4 weeks following TLPW acupuncture intervention. (a) Body weight (grammes). (b) Weight of the left kidney. (c) Kidney index. (d) Blood glucose level. (e) Triglyceride level. (f) Total cholesterol level. (g) Twenty-four-hour urinary protein level. (h) Serum creatinine level. (i) Blood urea nitrogen level. (j) Body weight change. (k) Blood glucose level change. (l) Urinary SPON2 level. Note: DKD + Acu group (*n* = 7), DKD group (*n* = 7), control group (*n* = 7); compared with the control group, ⁣^∗^*p* < 0.05; compared with the DKD group, #*p* < 0.05.

To investigate the impact of TLPW acupuncture, physicochemical parameters were measured in all groups. The body weight, kidney weight, kidney index and levels of blood glucose, TC, TG, 24-h urinary protein, urinary SPON2, BUN and SCR of DKD rats changed significantly compared to those of the control group (Figures 2a, 2b, 2c, 2d, 2e, 2f, 2g, 2h, 2i, 2j, 2k and 2l). We did not observe significant body weight disparities (Figure 2a,j), and a notable reduction was noted in both kidney weight (Figure 2b,k) and the kidney weight/body weight index (Figure 2c) within the DKD + Acu group versus the DKD group. TLPW acupuncture treatment significantly suppressed the increase in blood glucose and TG levels (Figure 2d,e), whereas the TC level was not greatly different between the DKD and DKD + Acu groups (Figure 2f). Moreover, we detected 24-h urinary protein, urinary SPON2, BUN and SCR levels to evaluate whether TLPW acupuncture has an effect on kidney injury in DKD rats. Increased 24-h urinary protein, urinary SPON2, BUN and SCR levels were observed in DKD rats, whilst this increase was significantly suppressed by TLPW acupuncture (Figures 2g, 2h, 2i and 2l).

### 3.3. TLPW Acupuncture Ameliorated Renal Pathology in DKD Rats

**Figure 3:**
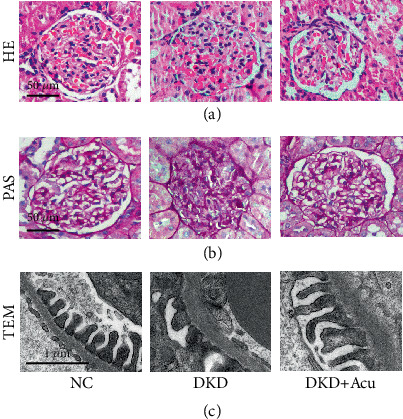
TLPW acupuncture induced measurable glomerular remodelling after 4 weeks. (a) HE staining (bars = 50 *μ*m). (b) PAS staining (bars = 50 *μ*m). (c) TEM (bars = 1 *μ*m).

To further evaluate the degree of kidney injury in DKD rats, HE, PAS staining and TEM were performed on renal tissues (Figure 3). The DKD group and DKD + Acu group showed glomerular capillary hypertrophy, mesangial matrix hyperplasia and basement membrane thickening. However, these features were absent in the control group. This indicated that DKD leads to different degrees of renal injury. However, better pathological manifestations were observed in the DKD + Acu group than in the DKD group (Figure 3a,b). Additionally, we found that DKD resulted in podocyte damage, which manifested as GBM thickening, and podocyte FPs demonstrated pathological alterations, including focal fusion and advanced effacement with loss of architectural integrity. However, compared to the DKD group, the fusion rate of the FPs decreased, the number of FPs increased and the basement membrane became thinner in the DKD + Acu group (Figure 3c).

### 3.4. Transcriptomic Analysis

**Figure 4:**
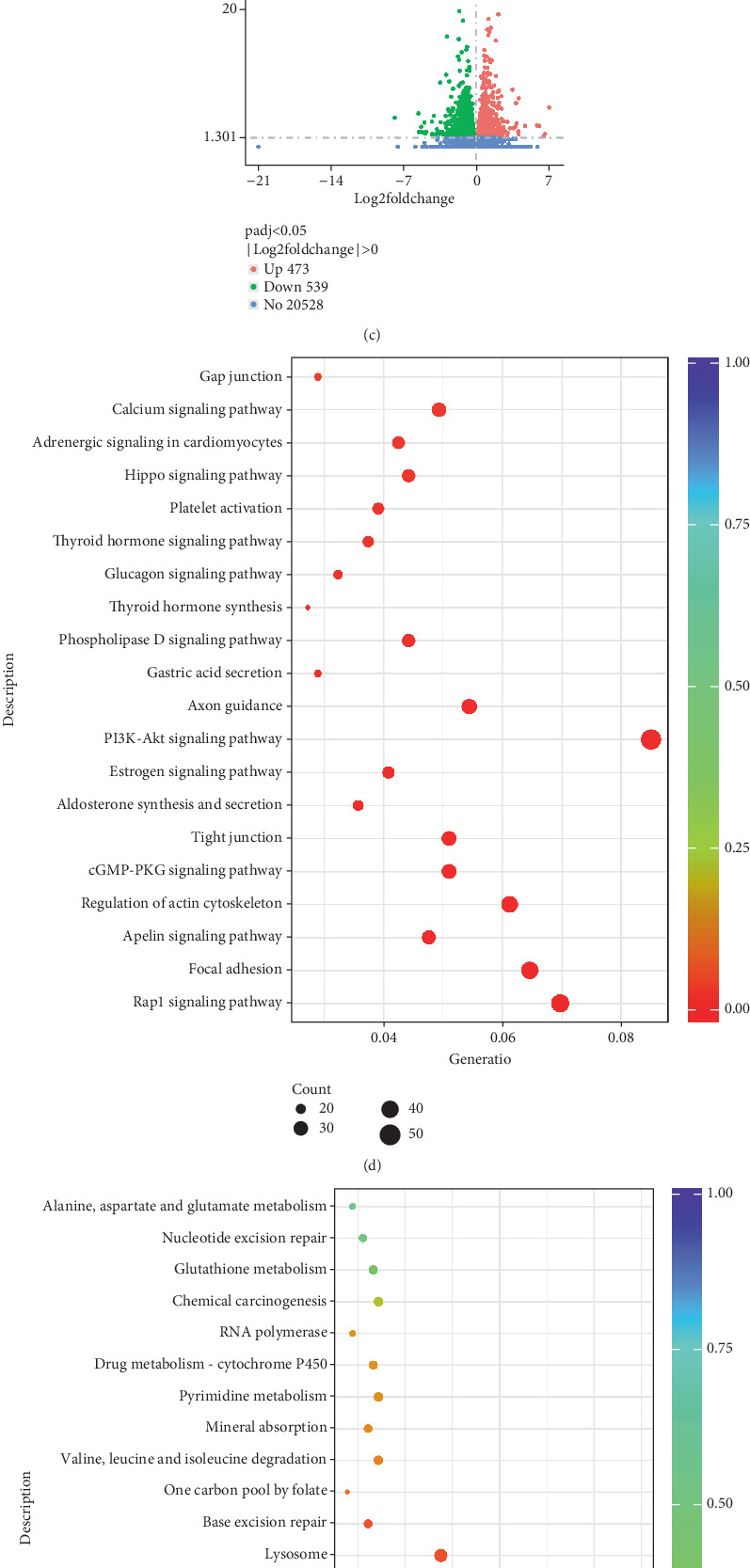
Data for the TLPW acupuncture treatment group, including differential gene expression, KEGG pathway analysis and GSEA results. (a) Analysis of differentially expressed genes (DEGs) between DKD (*n* = 5) and DKD + Acu (*n* = 5) groups. (b) Heatmap of genes in the NC (*n* = 5), DKD (*n* = 5) and DKD + Acu (*n* = 5) groups. (c) Analysis of DEGs between DKD (*n* = 5) and NC (*n* = 5) groups. (d) KEGG functional enrichment of downregulated genes between DKD (*n* = 5) and NC (*n* = 5) groups. (e) KEGG functional enrichment of upregulated genes between DKD (*n* = 5) and NC (*n* = 5) groups. (f) GSEA enrichment analysis between DKD (*n* = 5) and DKD + Acu (*n* = 5) groups. Note: AK: DKD + Acu group; MK: DKD group.

To identify key pathways and biological processes and screen therapeutic targets, transcriptomics and TMT-labelled proteomics were interrogated separately. There were 3275 DEGs in the DKD group versus the NC group, which consisted of 1630 upregulated and 1645 downregulated DEGs. Moreover, there were 1012 DEGs in the DKD + Acu group versus the DKD group, including 473 upregulated genes and 539 downregulated genes. The results are shown in Figures 4a, 4b and 4c. To further clarify the main target genes of TLPW acupuncture in reducing proteinuria in DKD rats, the DEGs between the DKD group and the NC group, as well as those between DKD + Acu and DKD groups, were subjected to KEGG or GSEA. The results are shown in Figures 4d, 4e and 4f. Significance thresholds were set at *p* adj < 0.05 and *q* < 0.2; KEGG analysis indicated that tight junction, focal adhesion, glucagon signalling pathway and gap junction were significantly downregulated in the DKD group, whilst other glycan degradation and proteasome were significantly upregulated. In addition, we performed GSEA of DKD and DKD + Acu groups and found that the composition of DKD + Acu was highly positively correlated with the insulin secretion (RNO04911), glucagon signalling (RNO04922) and adherens junction (RNO04520) pathways. Therefore, we speculated that TLPW acupuncture may regulate the metabolic function of glycolipid adherens junctions and inhibit the effect of EMT in the podocytes of DKD rats, thereby reducing proteinuria and podocyte injury.

### 3.5. TMT-Labelled Proteomics

**Figure 5:**
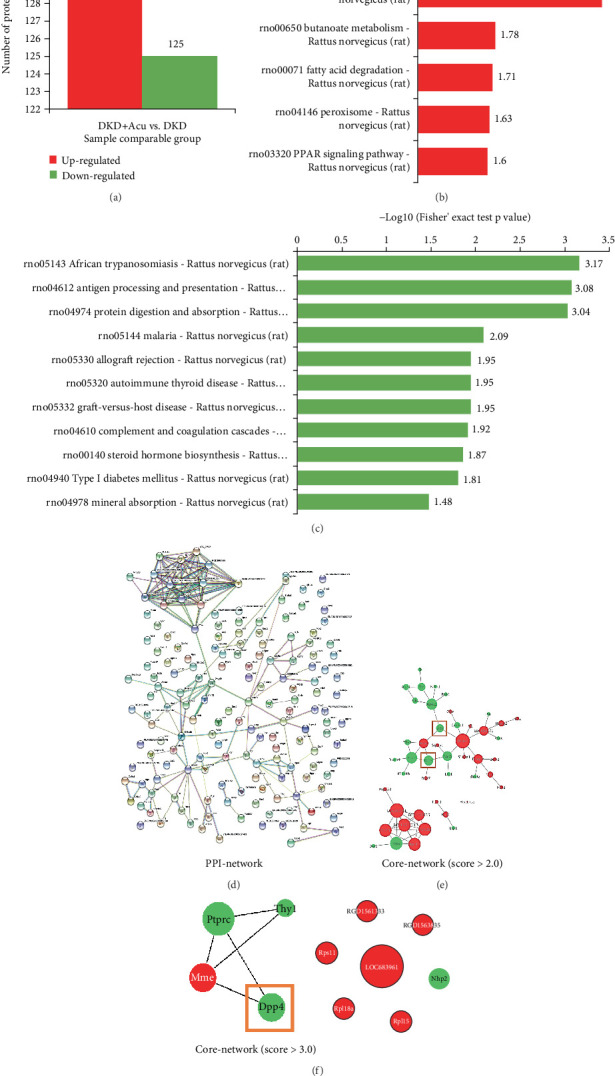
TMT-labelled proteomic analysis, KEGG pathway analysis and core target network construction. (a) Differentially expressed proteins between DKD (*n* = 3) and DKD + Acu (*n* = 3) groups. (b) Enrichment map of KEGG pathways for the upregulated proteins between DKD (*n* = 3) and DKD + Acu (*n* = 3) groups. (c) Enrichment map of KEGG pathways for the downregulated proteins between DKD (*n* = 3) and DKD + Acu (*n* = 3) groups. (d) PPI network of differentially expressed proteins. (e) The core network was drawn according to MCODE scores (score > 2.0). (f) Core genes were identified from the MCODE network (score > 2.0).

Three rat renal cortical tissue samples (six in total) were taken from DKD + Acu and DKD groups after 4 weeks of intervention. TMT quantitative proteomics in rat kidney tissue showed that a total of 5099 proteins were identified, of which 4351 contained quantitative information. Under the condition of a fold change > 1.2 as a significant upward regulation and fold change < 0.83 as a significant downward regulation whilst using a *t* test and *p* value < 0.05 as a criterion for significant change, 125 were downregulated, and 130 proteins were upregulated in DKD + Acu and DKD groups (Figure 5). Pathway annotations performed for differentially expressed proteins were enriched based on the KEGG database to obtain KEGG pathways for visual display. All the pathways involved in the genes encoding the differentially expressed proteins were obtained, and the significance level of each pathway was calculated using Fisher's exact test to predict 16 specific signalling pathways that were significantly involved in the differentially expressed proteins. The upregulated proteins were mainly involved in energy metabolism pathways of glycolipids; the downregulated proteins were mainly distributed in antigen recognition and expression and protein digestion and absorption-related pathways (Figure 5). The upregulated differentially expressed proteins were mainly distributed in ribosomal pathways, metabolic pathways, fatty acid metabolism, peroxisomes and PPAR signalling pathways. The downregulated differentially expressed proteins were mainly distributed in protein digestion, metabolic pathways, absorption pathways and antibody reaction and expression pathways. The results of the PPI network constructed by applying the http://STRING-db.org online tool are shown in Figure 5. Through the analysis of the MCODE plug-in using Cytoscape software, we found that DPP4 (Figure 5,f) had a high MCODE score [18, 19] in the regulation network, indicating its importance in the regulation network. Therefore, TLPW acupuncture was found to improve the energy metabolism of glycolipids and the protein expression of immune-related processes in DKD rats after 4 weeks of intervention, and DPP4 protein played an important role. Combined with transcriptomic analysis (2.3.5 Transcriptomic Analysis), these results revealed that DPP-4 enzymatically processes diverse peptide targets, such as brain natriuretic peptide (BNP), glucagon-like peptide-1(GLP-1), SDF-1*α* and neuropeptide Y, whilst SDF-1*α* can inhibit the expression of TGF-beta. We speculate that TLPW acupuncture ameliorates proteinuria in DKD rats by suppressing EMT via the DPP4/SDF-1*α*/TGF-*β*/Smad signalling axis.

### 3.6. TLPW Acupuncture Decreased Podocyte Injury and Ameliorated EMT in DKD Rats

**Figure 6:**
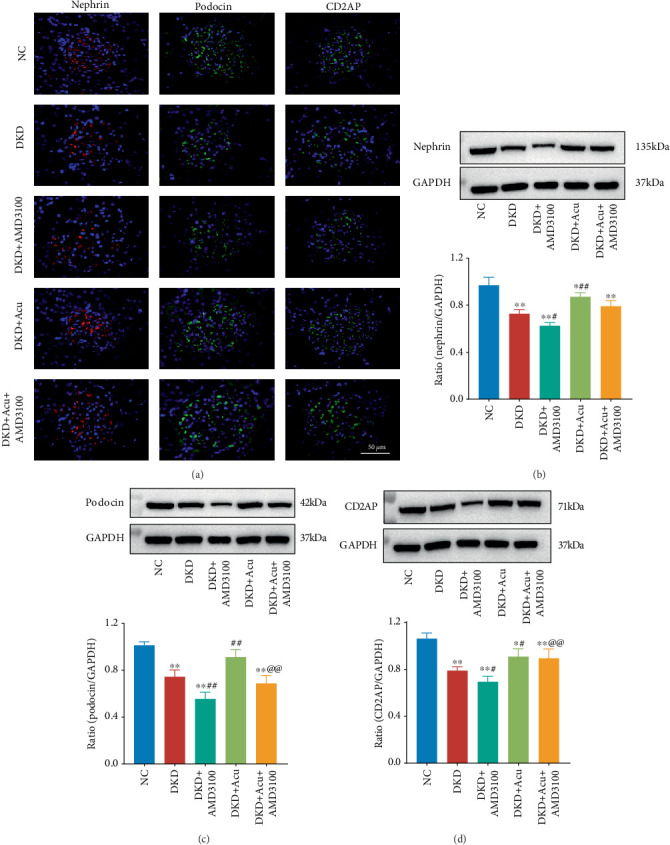
Analysis of podocyte-specific marker expression in the renal cortex via immunofluorescence staining and western blotting. (a) Immunofluorescence staining of podocyte-specific markers (CD2AP, podocin and nephrin) (bars = 50 *μ*m). (b) Western blot of analysis nephrin expression in the renal cortex. (c) Western blot analysis of podocin expression in the renal cortex. (d) Western blot analysis of CD2AP expression in the renal cortex. Note: ⁣^∗^*p* < 0.05,^∗∗^*p* < 0.01 versus control group; #*p* < 0.05, ##*p* < 0.01 versus DKD group; @*p* < 0.05, @@*p* < 0.01 versus DKD + Acu group.

To assess the presence of nephrin, podocin and CD2AP, which are specific markers for podocytes, IF was employed to examine the slit diaphragm between the FPs of podocytes in rats from each experimental group. The corresponding results can be observed in Figure 6a. In the NC group, the fluorescence intensity of podocyte-specific markers was the strongest, whilst that of the DKD, DKD + AMD3100, DKD + Acu and DKD + Acu + AMD3100 groups decreased greatly; however, versus the DKD group, the fluorescence intensity of podocyte-specific markers was enhanced in the DKD + Acu group, and there was a significant change between the DKD + Acu and DKD + Acu + AMD3100 groups. WB was also used to detect the relative expression of the above podocyte-specific markers, and the results are shown in Figures 6b, 6c, and 6d. The nephrin and CD2AP indexes of the DKD, DKD + AMD3100, DKD + Acu and DKD + Acu + AMD3100 groups were greatly decreased compared to those of the NC group (⁣^∗∗^*p* < 0.01 or ⁣^∗^*p* < 0.05). Compared with the DKD group, the DKD + Acu group greatly increased, and the DKD + AMD3100 group greatly decreased (##*p* < 0.01 or #*p* < 0.05). In the NC group, a decreased podocin index was observed in the DKD, DKD + AMD3100 and DKD + Acu + AMD3100 groups (⁣^∗∗^*p* < 0.01 or ⁣^∗^*p* < 0.05); nevertheless, the DKD + Acu group was not greatly changed. The significantly increased podocin index of the DKD + Acu group was observed (##*p* < 0.01) versus the DKD group, whilst the DKD + AMD3100 group significantly decreased (##*p* < 0.01), which indicated that TLPW acupuncture could increase the expression of the podocyte-specific marker molecules podocin and nephrin and alleviate podocyte injury. Amongst the above three indicators, we found a significant difference in the relative expression of podocin or CD2AP in the DKD + Acu group compared with the DKD + Acu + AMD3100 group (@@*p* < 0.01), suggesting that AMD3100 can reduce the acupuncture effect of TLPW acupuncture in improving podocyte injury.

**Figure 7:**
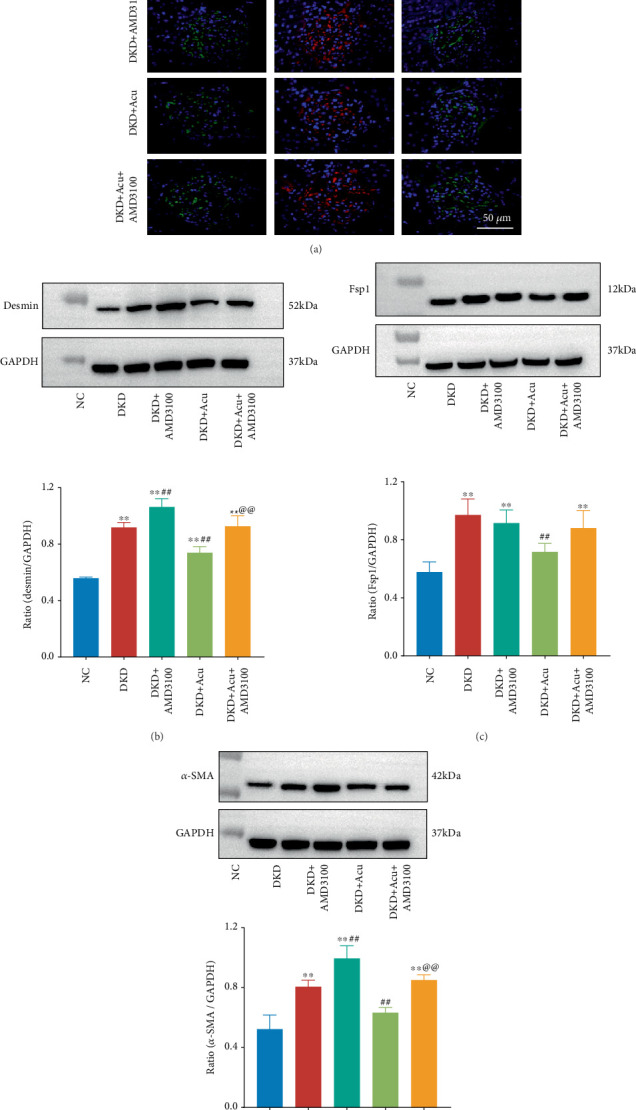
Analysis of epithelial–mesenchymal transition (EMT) marker expression in the renal cortex via immunofluorescence staining and western blotting. (a) Immunofluorescence staining of EMT markers (desmin, Fsp1 and *α*-SMA) (bars = 50 *μ*m). (b) Western blot analysis of desmin expression in the renal cortex. (c) Western blot analysis of Fsp1 expression in the renal cortex. (d) Western blot analysis of *α*-SMA expression in the renal cortex. Note: ⁣^∗^*p* < 0.05,^∗∗^*p* < 0.01 versus control group; #*p* < 0.05, ##*p* < 0.01 versus DKD group; @*p* < 0.05, @@*p* < 0.01 versus DKD + Acu group.

IF was used to detect the expression of desmin, Fsp1 and *α*-SMA, which are phenotypic markers of mesenchymal cells, in each group of rats (Figure 7a). Desmin, Fsp1 and *α*-SMA had the weakest fluorescence intensity in NC rats, whilst the fluorescence intensity was significantly increased in the DKD, DKD + AMD3100, DKD + Acu and DKD + Acu + AMD3100 groups, indicating that EMT rarely occurred in NC rats; however, compared with those in DKD rats, the fluorescence intensities of desmin, Fsp1 and *α*-SMA were decreased in DKD + Acu rats, and the results suggested that EMT was ameliorated in diabetic nephropathy rats after TLPW acupuncture intervention. WB was utilized to evaluate the relative expression of the above mesenchymal cell phenotypic markers (Figures 7b, 7c and 7d). Compared with the NC group, the DKD, DKD + AMD3100, DKD + Acu and DKD + Acu + AMD3100 groups exhibited a significantly increased desmin index (⁣^∗∗^*p* < 0.01), the DKD + Acu group showed a significant decrease versus the DKD group (##*p* < 0.01) and the DKD + AMD3100 group indicated a significant increase (##*p* < 0.01). Moreover, compared with the NC group, the DKD, DKD + AMD3100 and DKD + Acu + AMD3100 groups significantly increased the expression of Fsp1 and *α*-SMA (⁣^∗∗^*p* < 0.01), whilst the DKD + Acu group indicated great therapeutic effects versus the DKD group (##*p* < 0.01), which indicated that TLPW acupuncture inhibited the level of phenotypic markers of mesenchymal cells in DKD rats. Amongst the above three indicators, a significant difference in the relative expression levels of desmin and *α*-SMA between rats was observed in the DKD + Acu and DKD + AMD3100 + Acu groups (@@*p* < 0.01), indicating that AMD3100 could reduce the acupuncture effect of TLPW acupuncture in inhibiting the expression of phenotypic markers of mesenchymal cells.

### 3.7. The SDF-1*α* Receptor Antagonist AMD3100 Abolishes the Renal Protective Effect of TLPW Acupuncture via the DPP4/SDF-1*α*/TGF-*β*/Smad Signalling Axis

**Figure 8:**
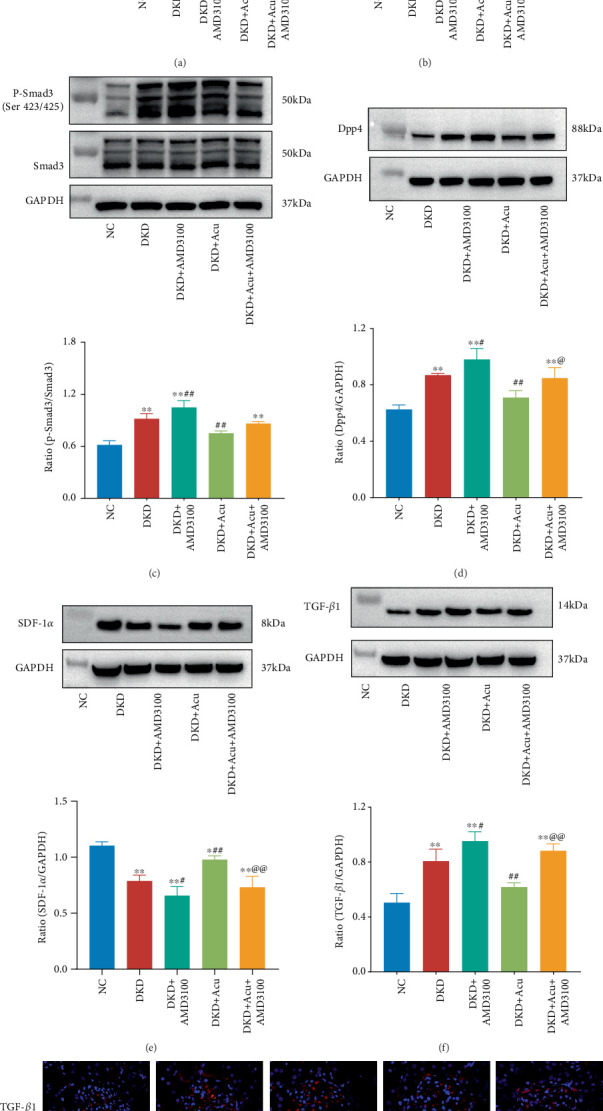
Analysis of DPP4/SDF-1*α*/TGF-*β*/Smad signalling activity in the renal cortex via immunofluorescence staining and western blotting. (a) Dpp-4 expression in the renal cortex. (b) Dpp-4 expression in serum. (c) Western blot analysis of the p-Smad3/Smad3 ratio in the renal cortex. (d) Western blot analysis of the DPP4 expression in the renal cortex. (e) Western blot analysis of SDF-1*α* expression in the renal cortex. (f) Western blot analysis of TGF-*β*1 expression in the renal cortex. (g) Immunofluorescence staining of TGF-*β*1 (bars = 50 *μ*m). ⁣^∗^*p* < 0.05,^∗∗^*p* < 0.01 versus control group; #*p* < 0.05, ##*p* < 0.01 versus DKD group; @*p* < 0.05, @@*p* < 0.01 versus DKD + Acu group.

To determine the role of the DPP4/SDF-1*α*/TGF-*β*1/Smad signalling axis in the mechanism of TLPW acupuncture, an intraperitoneal injection of the receptor antagonist of SDF-1*α* AMD3100 was administered. The concentration of Dpp-4 in the renal cortex and serum of rats in each group was quantified by enzyme-linked immunosorbent assay (Figure 8). Following a 4-week TLPW acupuncture intervention, versus the NC group, significantly increased renal cortex and serum levels of Dpp-4 in the DKD, DKD + AMD3100, DKD + Acu and DKD + Acu + AMD3100 groups were observed (⁣^∗^*p* < 0.05 or ⁣^∗∗^*p* < 0.05). The Dpp-4 content in the DKD + Acu group was greatly alleviated compared with that in the DKD group (#*p* < 0.05). AMD3100 inhibited the acupuncture effect of TLPW acupuncture and increased the Dpp-4 of renal cortex content in the DKD + Acu + AMD3100 group, greatly differing from that in the DKD + Acu group (@@*p* < 0.01). WB detected DPP4/SDF-1*α*/TGF-*β*/Smad signalling pathway protein levels in the rat renal cortex (Figure 8). Following a 4-week TLPW acupuncture intervention, not only the relative expression of DPP4 and Tfg*β*1 protein but also the p-Smad3/Smad3 index versus NC, DKD, DKD + AMD3100 and DKD + Acu + AMD3100 groups greatly increased (⁣^∗∗^*p* < 0.01), whilst the above three proteins in the DKD + Acu group showed no statistical significance (*p* > 0.05). The DPP4 and TGF-*β*1 proteins as well as the p-Smad3/Smad3 index in the DKD + Acu renal cortex decreased (##*p* < 0.01), and the DPP4 and TGF-*β*1 indicators in the DKD + AMD3100 renal cortex were significantly different from the DKD group (#*p* < 0.05). The DKD, DKD + Acu, DKD + AMD3100 and DKD + Acu + AMD3100 groups exhibited a significant change in the SDF-1*α* index (⁣^∗∗^*p* < 0.01 or ⁣^∗^*p* < 0.05) versus the NC group, whilst versus the DKD group, both the DKD + Acu and DKD + AMD3100 groups showed significant improvement or inhibition (##*p* < 0.01 or #*p* < 0.05). However, DPP4, SDF-1*α* and TGF-*β*1 protein levels in the renal cortex were greatly different between the DKD + Acu and DKD + Acu + AMD3100 groups (@*p* < 0.05 or @@*p* < 0.01). The expression of TGF-*β*1 was detected by IF in each group (Figure 8), and the fluorescence intensity of TGF-*β*1 in the DKD, DKD + AMD3100, DKD + Acu and DKD + Acu + AMD3100 groups was enhanced versus the NC group; however, the fluorescence intensity in the DKD + Acu group was attenuated versus the DKD group. The results above revealed that TLPW acupuncture could block Smad3 phosphorylation and inhibit TGF-*β* signal transduction by reducing SDF-1*α* degradation and inhibiting Dpp-4 activity, whilst AMD3100 could abolish the renoprotective effect of TLPW acupuncture via the DPP4/SDF-1*α*/TGF-*β*/Smad signalling axis.

## 4. Discussion

DKD is a progressive microvascular complication of diabetes caused by glomerular capillary injury. A decreased glomerular filtration rate or persistent proteinuria is the main clinical manifestation [20]. At present, the general treatment regimen for hospitalized DKD patients is to control hyperglycaemia, control blood pressure and use ACEIs or ARBs, which have limited effects, cannot effectively prevent the progression of DKD and have the risk of further damage to the liver and kidney [21–23]. Chinese medicine plays a significant role in treating DKD. Acupuncture, as an effective complementary and alternative therapy, has been applied in 183 countries and regions [24]. There are numerous randomized controlled trials affirming the effectiveness of acupuncture in managing metabolic disorders [25]. We further explored the clinical and basic experiments of previous TLPW acupuncture. TLPW acupuncture is dominated by Zhongwan (RN12), Zusanli (ST36) and Yinlingquan (SP9), which can smooth the qi activity in the middle jiao and tonify the spleen and stomach to make clear yang rise and cloudy yin fall. Xuehai (SP10), Sanyinjiao (SP6) and Diji (SP8) function as ministerial acupoints; they can remove stasis in blood, give birth to new blood and restore the source of generation and operationalization. In addition, Sanyinjiao (SP6) can nourish blood and nourish yin, Yinlingquan (SP9) plays a role in invigorating the spleen and unblocking the waterway, Xuehai (SP10) can lead blood to the spleen and Diji (SP8) is good at promoting blood circulation and nourishing blood. With Quchi (LI11), Taichong (LR3), Fenglong (ST40) and Hegu (LI4) as adjuvant acupoints, Quchi (LI11) coordinates the stomach and intestines and harmonizes the stomach for descending adverse qi, Hegu (LI4) gives full play to the two-way effect of rising and falling and Hegu (LI4) and Quchi (LI11) can be used together to descend the stomach and intestine and cleanse impurities. Fenglong (ST40) plays the role of resolving dampness, eliminating phlegm and moistening the intestine. Taichong (LR3) is good at calming the liver and tonifying the spleen and demonstrates significant efficacy in diabetes and its complications. Previous studies have demonstrated that acupuncture at these acupoints ameliorates DKD through mechanisms such as regulating renal ferroptosis [26], improving insulin secretion for glycaemic control [27], modulating renal blood flow [28] and alleviating inflammatory responses [29], though whether these effects involve the DPP4/SDF-1*α*/TGF-*β*/Smad signalling axis remains unclear.

In this research, after STZ-induced DKD was successfully modelled in rats, we found greatly higher 24-h urinary protein levels, blood lipid and fasting blood glucose versus the control group, and pathological phenomena of renal injury, such as basement membrane thickening, glomerular capillary hypertrophy and mesangial matrix proliferation, were observed. With the intervention of TLPW acupuncture, blood lipid, 24-h urine protein, urea nitrogen levels, SCR and kidney organ coefficients were significantly decreased, and with the extension of acupuncture time, biochemical indicators such as blood lipid, urea nitrogen levels, SCR, 24-h urine protein and kidney organ coefficients in the DKD + Acu group were still greatly lower than in successfully modelled rats; moreover, the pathological manifestations of DKD were alleviated. These results suggested that there is a severe glomerular filtration barrier injury in DKD rats. TLPW acupuncture has a protective effect on renal damage in DKD, and continuous acupuncture treatment can play a beneficial role in managing the disease. Then, we performed transcriptomic and proteomic screenings of key pathways and targets and found that they were closely related to tight junction, the glucagon signalling pathway, gap junction, the cell adhesion molecule pathway and the DPP4 protein. As terminally differentiated epithelial cells, podocytes are also central targets for the development of proteinuria in DKD, and podocyte FP fusion, hypertrophy, shedding, loss and necrosis, which are early manifestations of injury, are the cellular basis for the progression of DKD [30]. Podocyte transdifferentiation (EMT) is one of the manifestations of podocyte injury and is mainly identified by downregulation of the nephrin and WT1 and upregulation of the mesenchymal cell markers desmin and *α*-SMA [31]. In the process of DKD, many factors, such as oxidative stress, inflammatory response and autophagy dysregulation, can lead to podocyte injury and the formation of proteinuria [4] and are associated with multiple signalling pathways, such as the Wnt/*β*-catenin pathway, TGF-*β*/Smad pathway and ILK pathway [32]. It has also been confirmed that the canonical Smad pathway and many other pathways are relevant to the induction of the podocyte EMT process and the mutual interference and interaction between the pathways cause the mechanism of podocyte EMT more complex [33]. At present, it is believed that the development of diabetic nephropathy can be attributed to elevated levels of glucose and lipid metabolism disorders and haemodynamic changes, cytokines, inflammatory response, oxidative stress, genetic susceptibility and other factors. Previous studies have suggested that TGF-*β* is a major mediator of EMT induction [34]. Previous studies have indicated that EMT can be induced by TGF-*β* stimulation alone in epithelial lineage cells cultured in vitro and the TGF-*β* concentration is positively correlated with the podocyte injury degree [35]. Therefore, the TGF-beta signalling pathway was selected as a target pathway of TLPW acupuncture-mediated podocyte transdifferentiation in rats with DKD for further mechanistic investigation.

Nephrin, podocin and CD2AP are representative molecules that constitute the podocyte slit diaphragm (SD), and their abnormal expression can affect the integrity of the pore size barrier to varying degrees and is closely related to the occurrence of proteinuria. Amongst them, nephrin is a major component of the podocyte slit diaphragm, and its lesions not only compromise the effect of the filtration membrane but also cause early podocyte dysfunction through EMT, prompting increased extracellular matrix secretion and leading to glomerular sclerosis and fibrosis [36]. Podocin can interact with nephrin and CD2AP to form the nephrin complex, and CD2AP directly interacts with nephrin both in vitro and in vivo, functioning as a structural bridge that mediates the association between nephrin and podocin. EMT in podocytes can lead to the destruction of their structure, cytoskeletal rearrangement, disappearance of podocytic processes and slit diaphragms and increased expression of mesenchymal cell phenotypic markers including *α*-SMA, Fsp1 and desmin.

Desmin is an intermediate filament protein abundantly expressed after podocyte injury, leading to podocyte cytoskeletal rearrangement, and is usually highly expressed in glomerular diseases of podocyte injury [37]. *α*-SMA is one of the marker proteins in the development of EMT in epithelial cells stimulated by TGF-*β*, and the degree of podocyte injury can be identified and indirectly reflects the degree of renal fibrosis by detecting the expression of *α*-SMA [38]. Fsp1 can be detected in large amounts after renal fibrosis and is basically undetectable in normal renal tissues [39]. The results showed downregulated protein expression of nephrin, podocin and CD2AP and upregulated protein expression of desmin, *α*-SMA and Fsp1 in the renal cortex of DKD group rats, with desmin and *α*-SMA being the most significantly upregulated. However, the protein expression of podocin and nephrin was upregulated, and the protein levels of desmin, *α*-SMA and Fsp1 was downregulated in the DKD + Acu group, indicating that TLPW acupuncture can reduce podocyte injury and thus inhibit EMT progression.

As an integral Type II transmembrane glycoprotein, Dpp-4 can cleave many cytokines and chemokines, such as SDF-1 [40]. SDF-1, however, has multiple isoforms, such as SDF-1*α*, SDF-1*β* and SDF-1*γ*, of which SDF-1*α* is the main isoform [41]. However, SDF-1*α* requires binding to its specific receptor CXCR4 to act, whilst selective blockade of the SDF-1*α* receptor aggravates podocyte loss [42] and increasing SDF-1*α* levels in the kidney and plasma is beneficial for alleviating proteinuria in DKD [43]. Therefore, in this study, we used AMD3100, an antagonist of the SDF-1*α* receptor CXCR4, to block the interaction between CXCR4 and SDF-1*α* and investigated whether TLPW acupuncture can inhibit SDF-1*α* degradation by inhibiting Dpp-4. We found higher SDF-1*α* levels in the DKD + Acu group than in the model group following the 4-week TLPW acupuncture intervention, which was attributed to the inhibition of Dpp-4 activity by TLPW acupuncture. This suggests that TLPW acupuncture can exert its renoprotective effect by reducing SDF-1*α* degradation and inhibiting Dpp-4 activity in rats with DKD. TGF-*β*1 and p-Smad3/Smad3 expression levels were also measured in each group, and we found significantly decreased p-Smad3/Smad3 and TGF-*β*1 levels in the DKD + Acu group versus the model group, indicating that DKD could induce TGF-*β*/Smad3 signalling pathway activation, but TLPW acupuncture inhibited TGF-*β*/Smad 3 signal transduction. After the intervention with AMD3100, we found increased p-Smad3 and TGF-*β* levels in the DKD + Acu + AMD3100 group versus DKD + Acu group, indicating that AMD3100 attenuated the acupuncture effect of TLPW acupuncture to inhibit TGF-*β*/Smad3 signal transduction.

## 5. Conclusions

According to the bioinformatics analysis results of the transcriptomics and proteomics, we speculate that TLPW acupuncture ameliorates proteinuria in DKD rats by suppressing EMT via the DPP4/SDF-1*α*/TGF-*β*/Smad signalling axis, which was validated in further animal experiments. Our study found that TLPW acupuncture could ameliorate glucose and lipid metabolism disorders, protect renal structure and reduce renal injury in DKD rats. In addition, TLPW acupuncture improved podocyte EMT in DKD rats by upregulating podocyte-specific marker (nephrin and podocin) levels and inhibiting mesenchymal cell phenotypic marker (desmin, *α*-SMA and Fsp1) levels. By using the SDF-1*α* receptor CXCR4 antagonist AMD3100 for related mechanistic experiments, it was found that TLPW acupuncture can inhibit the activity of Dpp-4 and reduce SDF-1*α* degradation, thereby inhibiting TGF-*β*1/Smad3 signalling pathway activation, improving podocyte EMT and reducing proteinuria in DKD rats. That is, TLPW acupuncture can attenuate EMT-induced podocyte injury and reduce proteinuria in DKD rats by regulating the Dpp-4/SDF-1*α*/TGF-*β* signalling axis and reducing proteinuria, thereby reducing renal injury.

## Data Availability

The data used to support the findings of this study are included within the article. The data used to support the findings of this study are available from the corresponding authors upon request.

## Nomenclature

ACRIs: angiotensin-converting enzyme inhibitors
AGC: automatic gain control
BUN: blood urea nitrogen
ARBs: angiotensin receptor blockers
DEGs: differentially expressed genes
DKD: diabetic kidney disease
ESRD: end-stage renal disease
EMT: epithelial–mesenchymal transition
FDR: false discovery rate
KEGG: Kyoto Encyclopedia of Genes and Genomes
GO: Gene Ontology
TCM: traditional Chinese medicine
SCR: serum creatinine
TC: total cholesterol
TG: triglyceride
UACR: urinary protein–creatinine ratio
eGFR: estimated glomerular filtration rate
24-hUpro: 24-h urinary protein content

## Supporting Information

Supporting Information 1 Haematoxylin and eosin (HE) staining.

Supporting Information 2 Periodic acid–Schiff (PAS) staining.

Supporting Information 3 Transmission electron microscopy (TEM).

Supporting Information 4 Western blot.

Supporting Information 5 The ARRIVE guidelines.
