# Supplementary figures and images for: Transcriptomics and Proteomics Reveal That TLPW Acupuncture Ameliorates Proteinuria in Diabetic Kidney Disease Model Rats by Suppressing Epithelial-to-Mesenchymal Transition via the DPP4/SDF-1α/TGF-β/Smad Signalling Axis
Source: J Diabetes Res. 2025 Oct 1;2025:2379872. doi: 10.1155/jdr/2379872 (PMC12507495; doi:10.1155/jdr/2379872)

Control group(n=3)


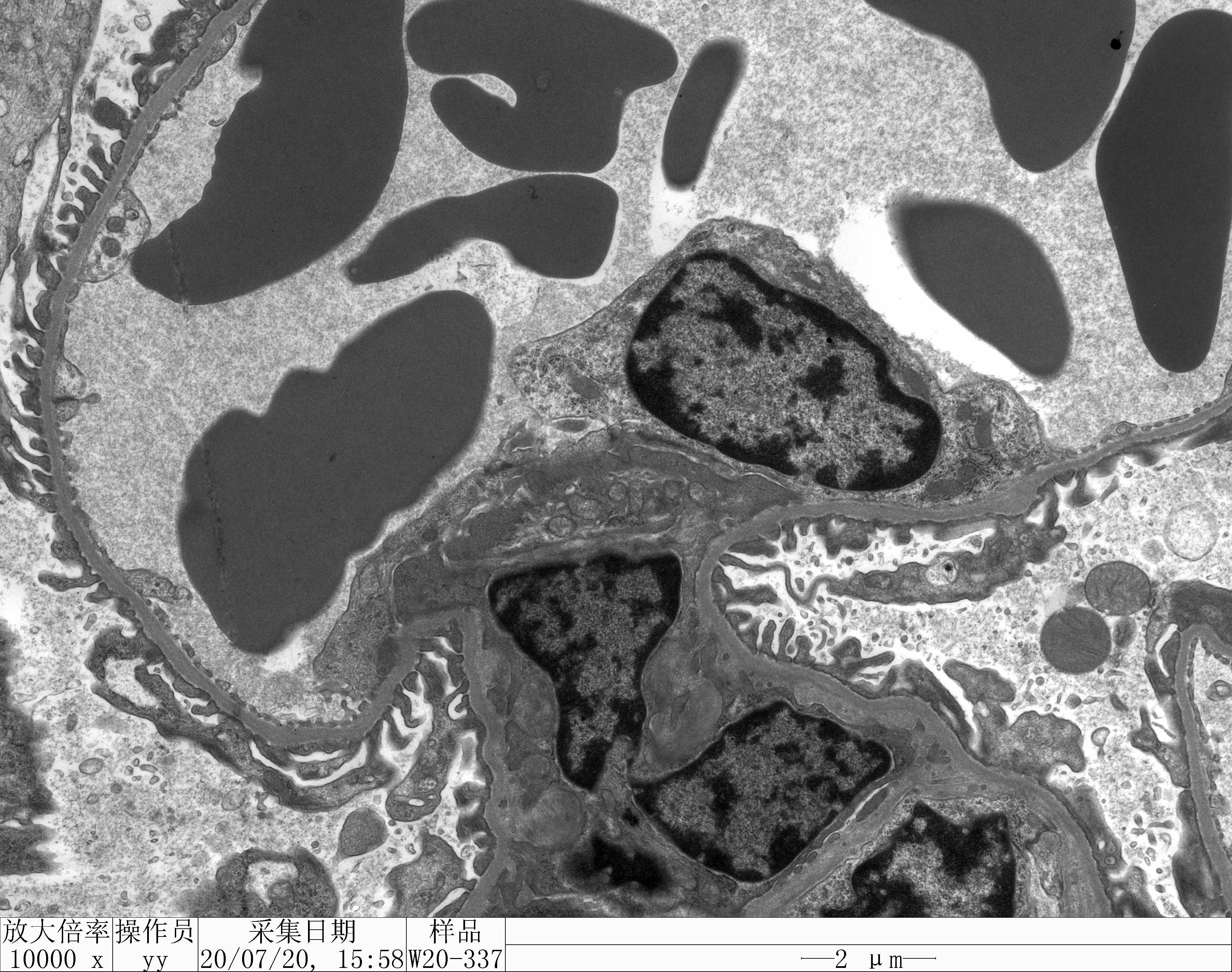


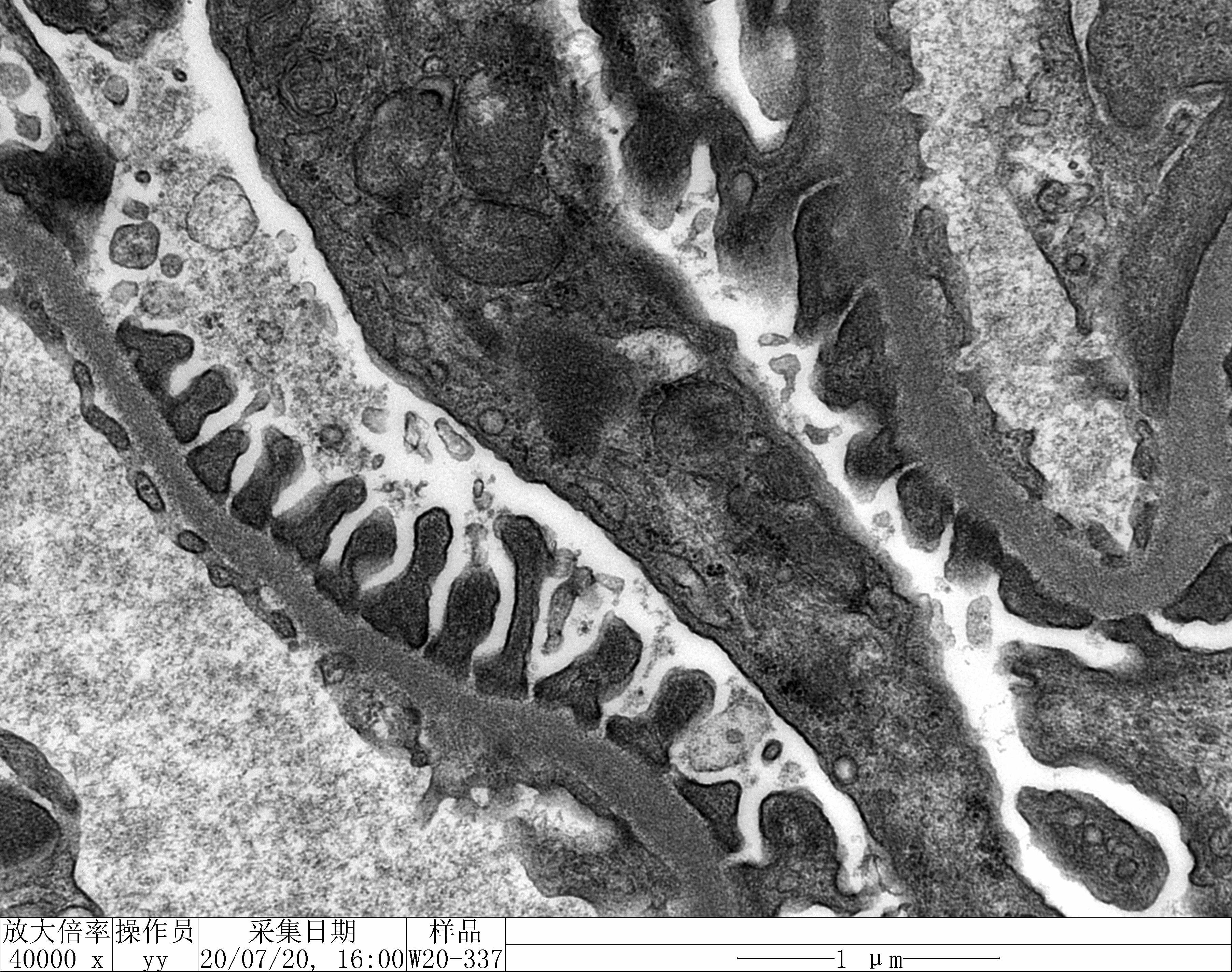


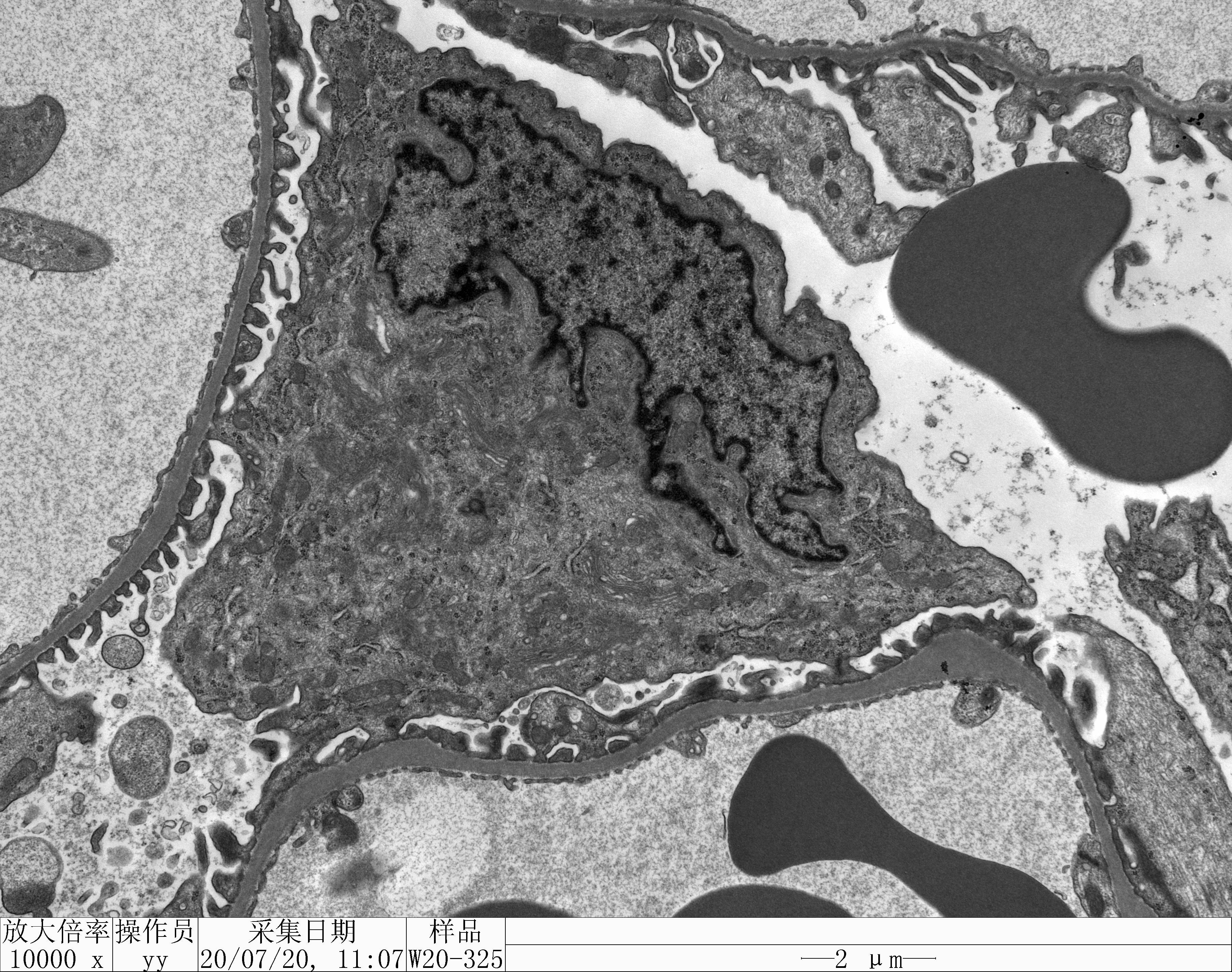


DKD group(n=3)


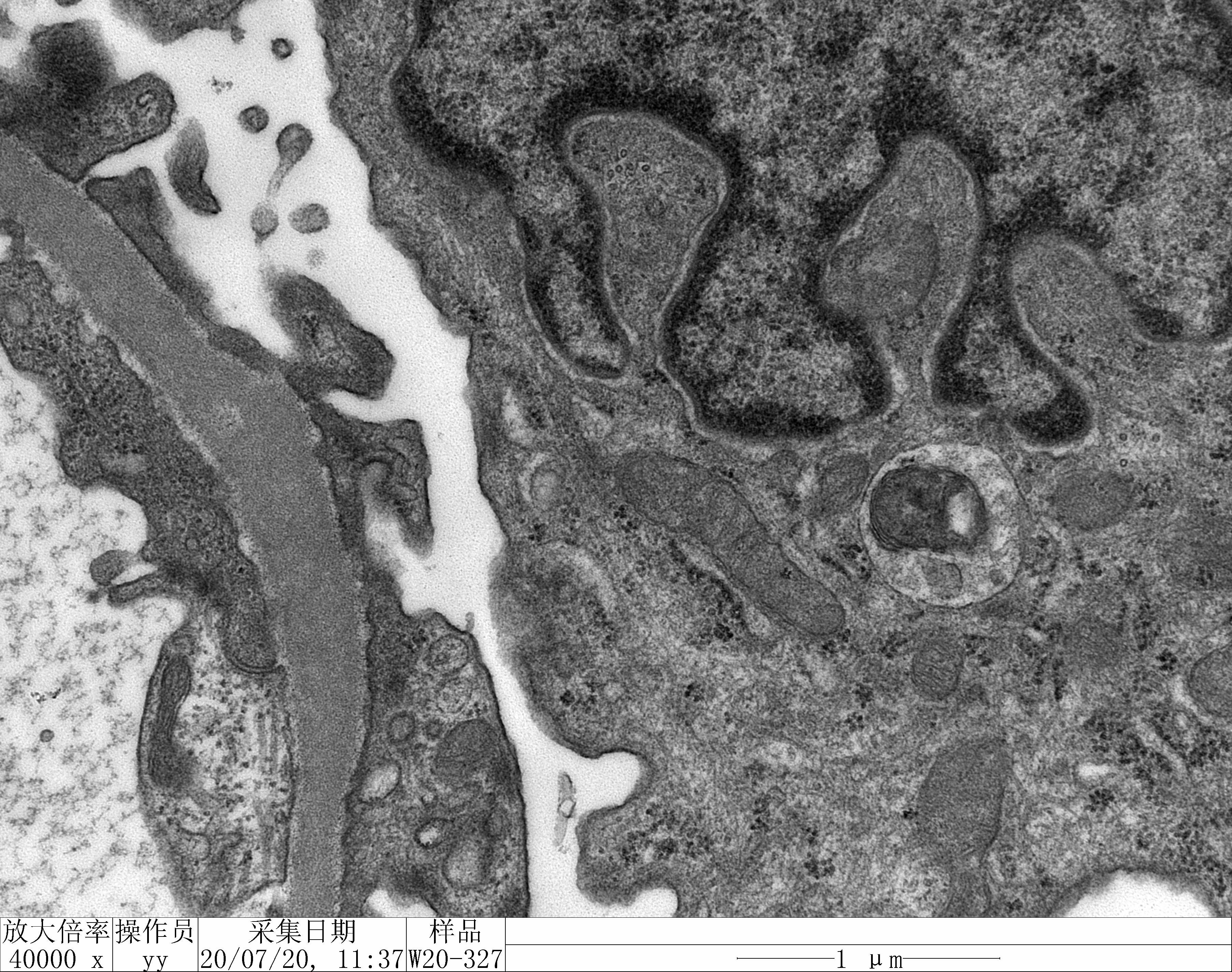


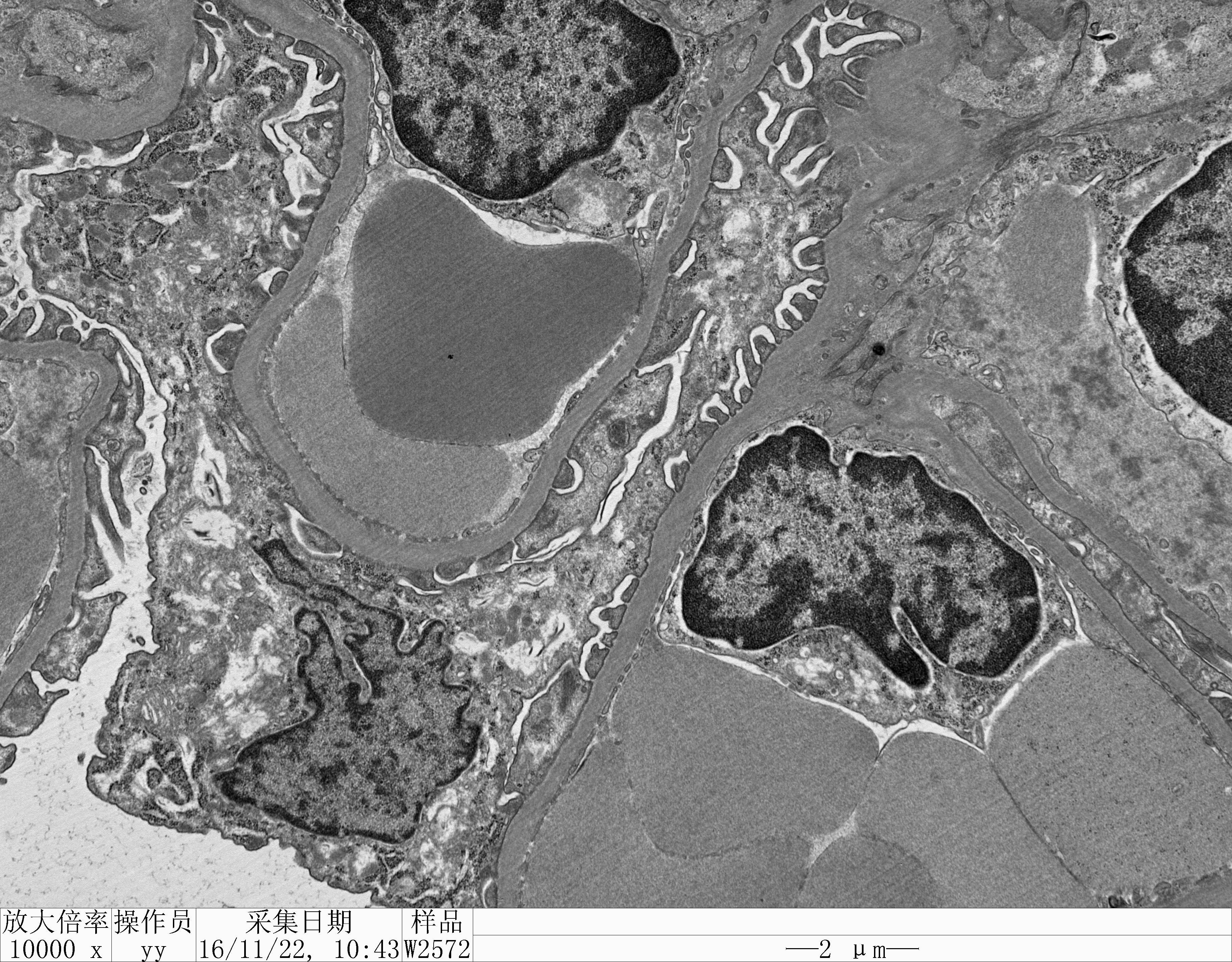


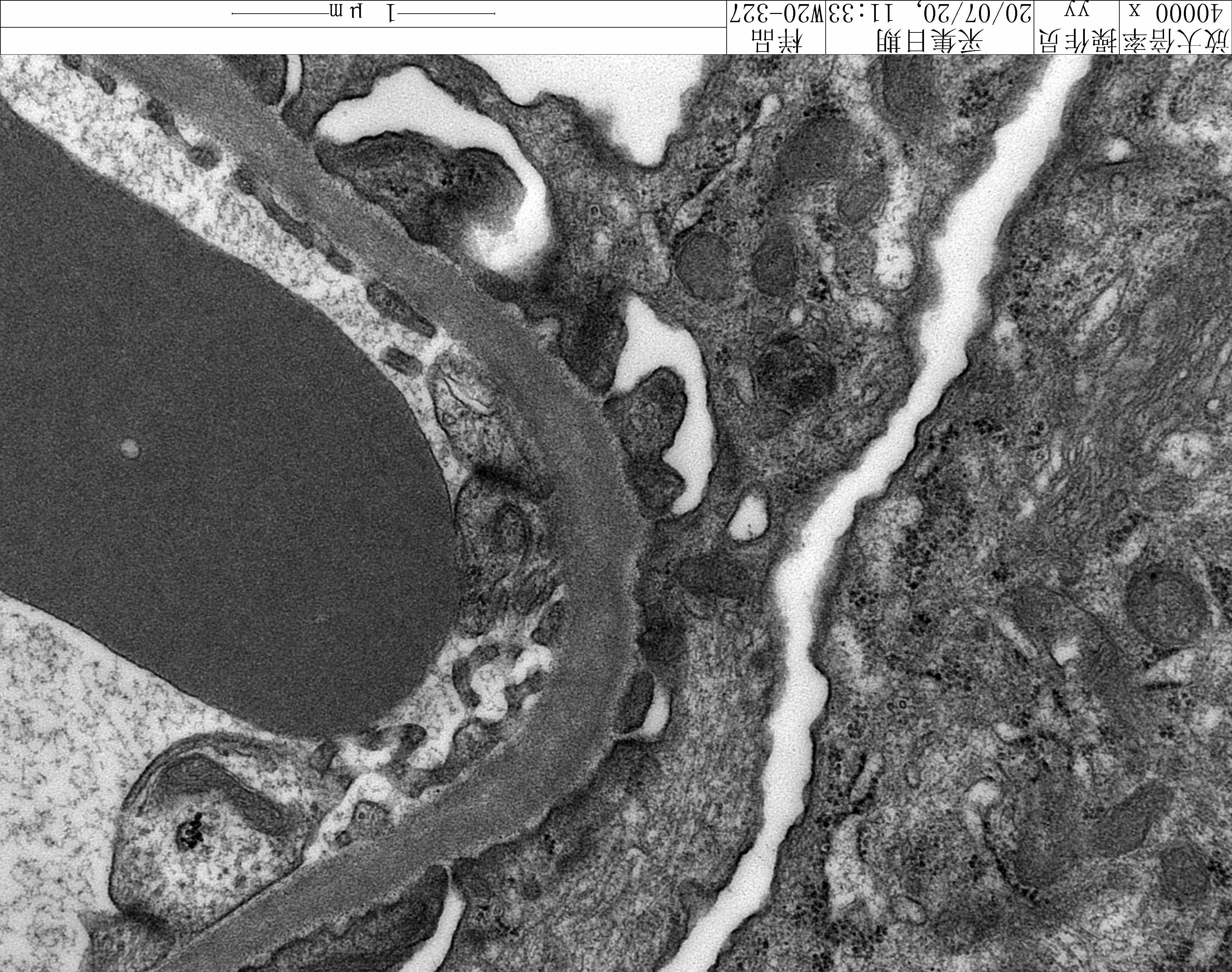


DKD+ACU group(n=3)


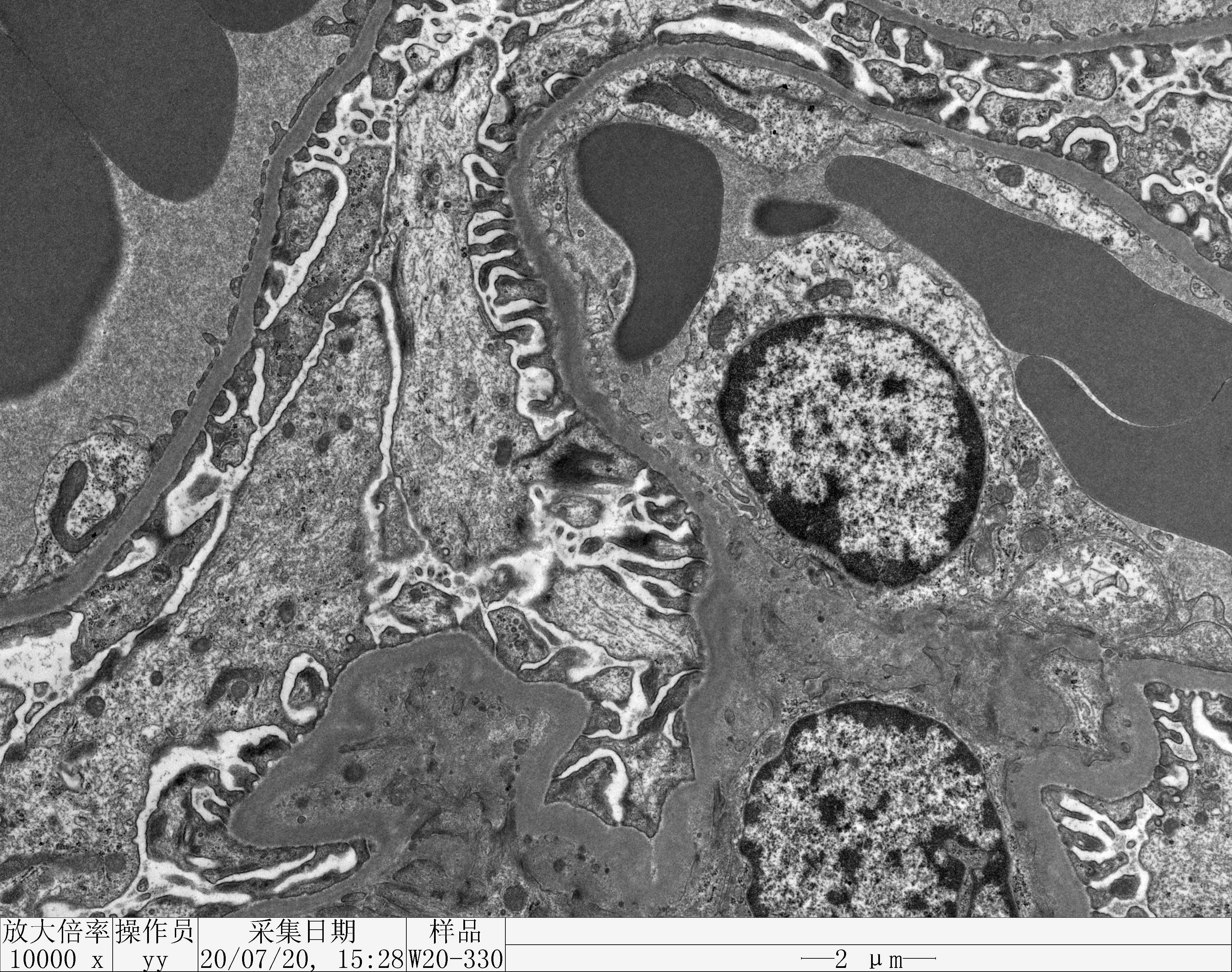


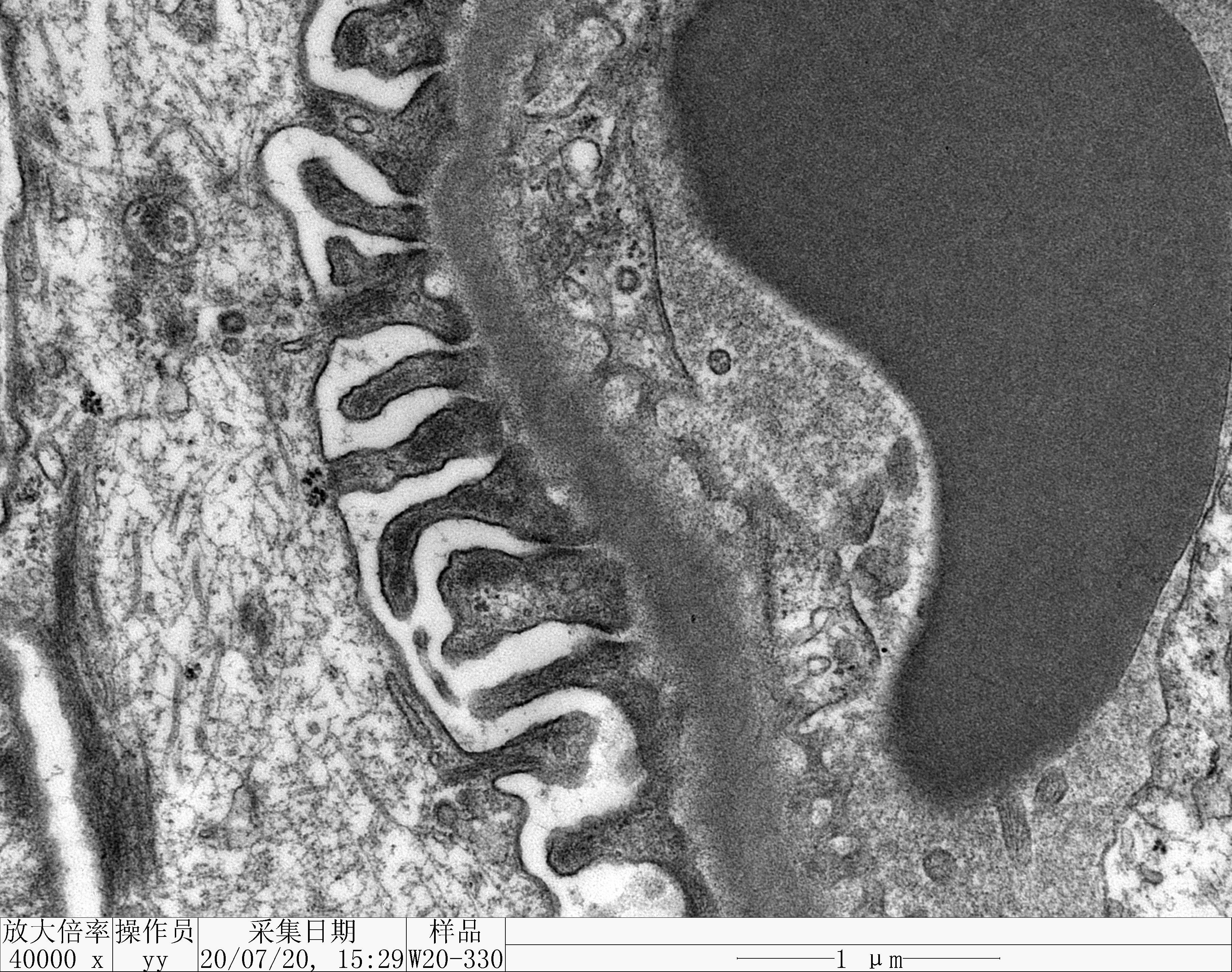


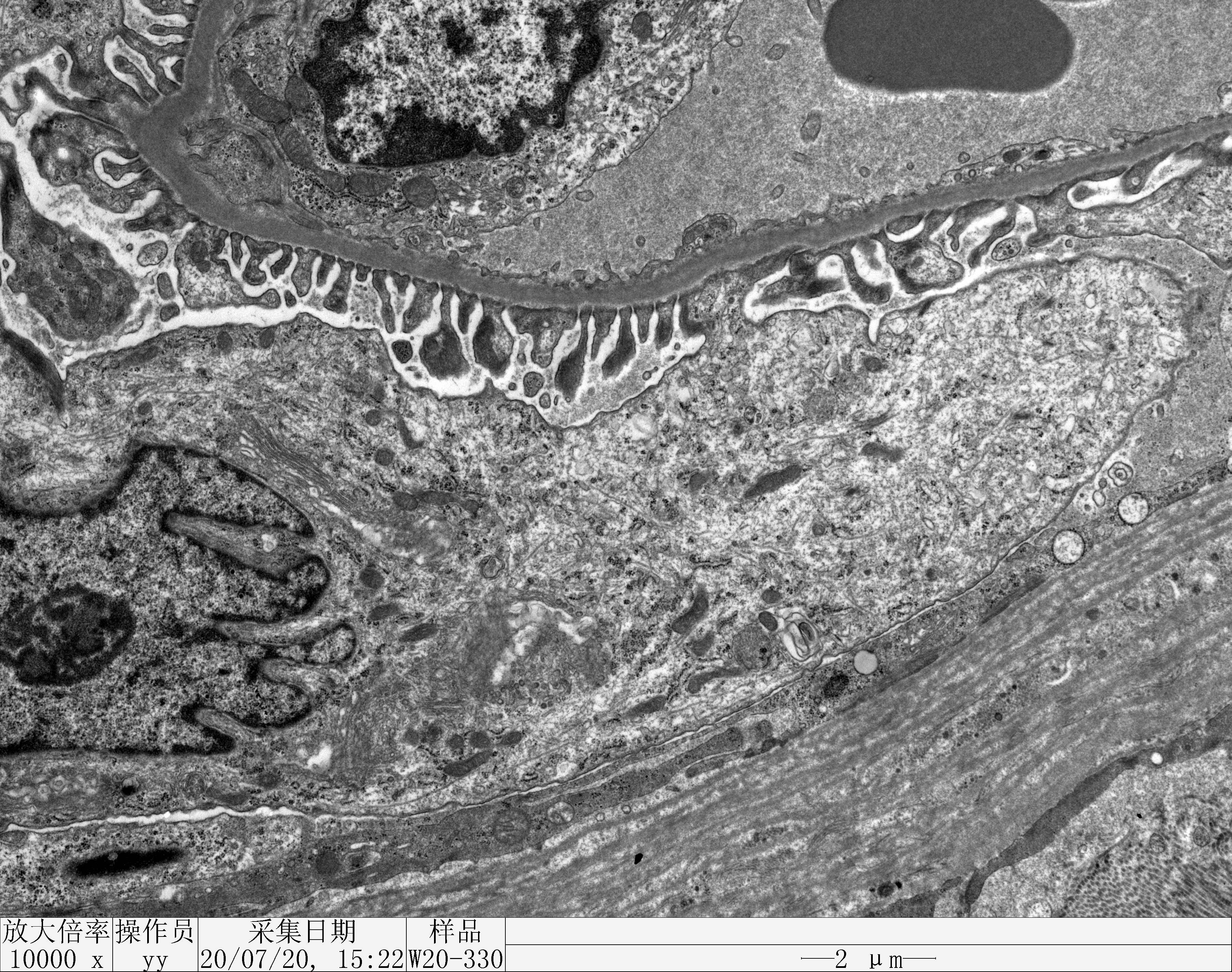

Supplement: Supporting Information 3 — Transmission electron microscopy (TEM). [file 2379872.f3.doc]
