# Supplementary material for: Transcriptomics and Proteomics Reveal That TLPW Acupuncture Ameliorates Proteinuria in Diabetic Kidney Disease Model Rats by Suppressing Epithelial-to-Mesenchymal Transition via the DPP4/SDF-1α/TGF-β/Smad Signalling Axis
Source: J Diabetes Res. 2025 Oct 1;2025:2379872. doi: 10.1155/jdr/2379872 (PMC12507495; doi:10.1155/jdr/2379872)

Western Blot

CD2AP	Rabbit	CST	#2135	1:1000
Desmin	Rabbit	CST	#5332	1:1000
Dpp4	Rabbit	CST	#61408	1:1000
Fsp1	Rabbit	Proteintech	20886-1-AP	1:800
Nephrin	Rabbit	Affinity	DF7501	1:500
Podocin	Rabbit	Proteintech	20384-1-AP	1:800
p-Smad3	Rabbit	CST	#9520	1:500
SDF-1α	Rabbit	Novus	NBP2-29480	1:1000
Smad3	Rabbit	CST	#9513	1:1000
TGF-β1	Rabbit	Abcam	ab215715	1:1000
α-SMA	Rabbit	CST	#19245	1:1000
GAPDH(37KD)	Mouse	优抗	UM4002	1：2000
goat anti-rabbit IgG-HRP	Goat	Affinity	S0001	1：4000
anti-mouse IgG-HRP	Goat	Affinity	S0002	1：4000

Marker

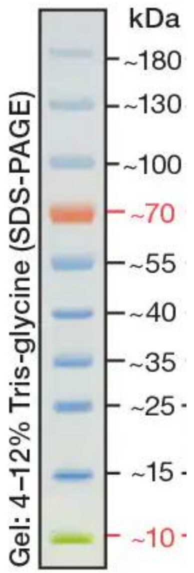

CD2AP 71kd

1~2 repeat

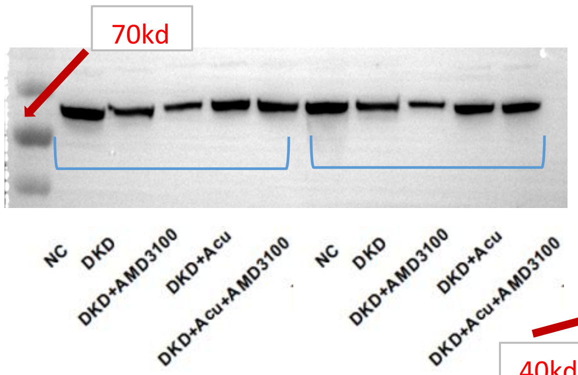

GAPDH(37KD)

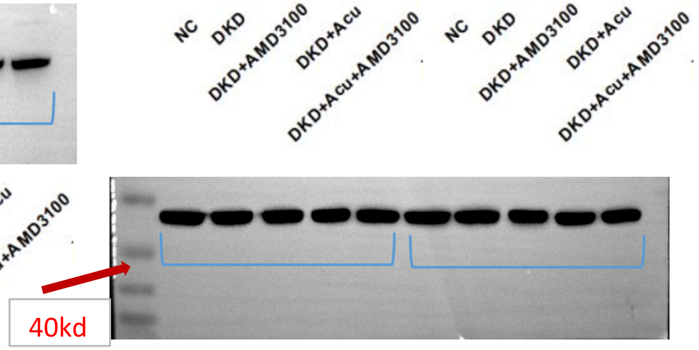

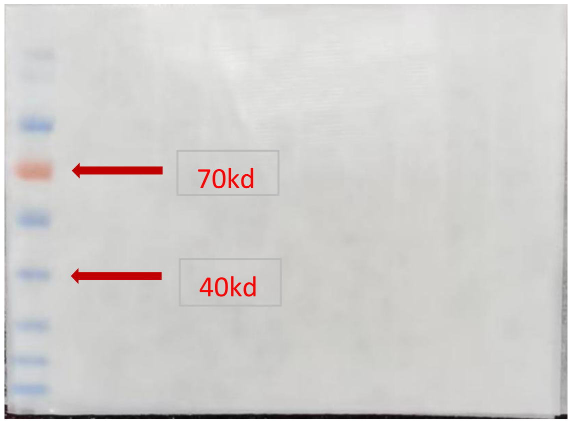

3repeat

GAPDH(37KD)

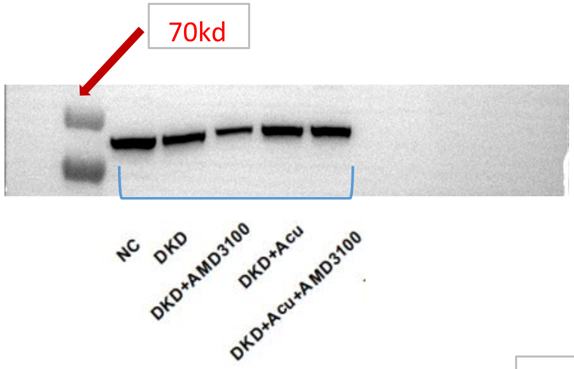

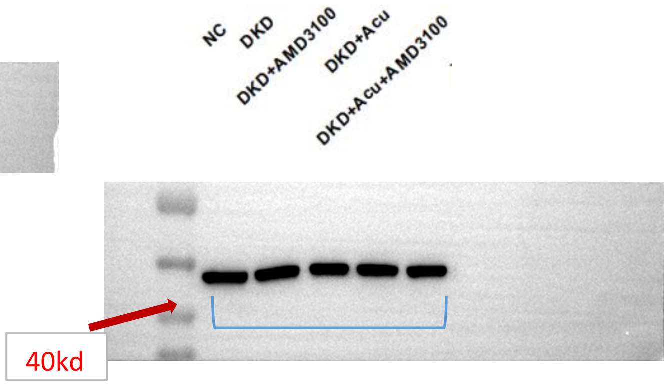

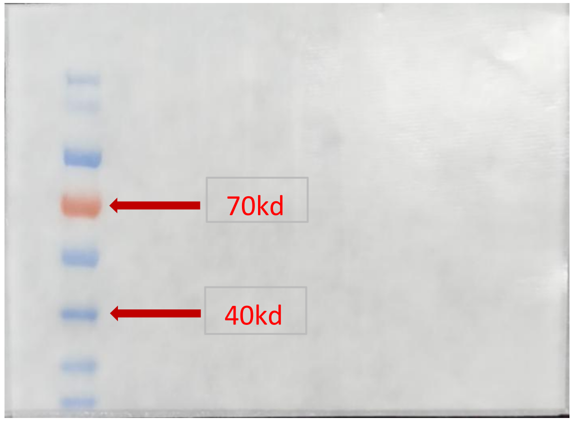

Desmin 52kd

1~2 repeat

GAPDH(37KD)

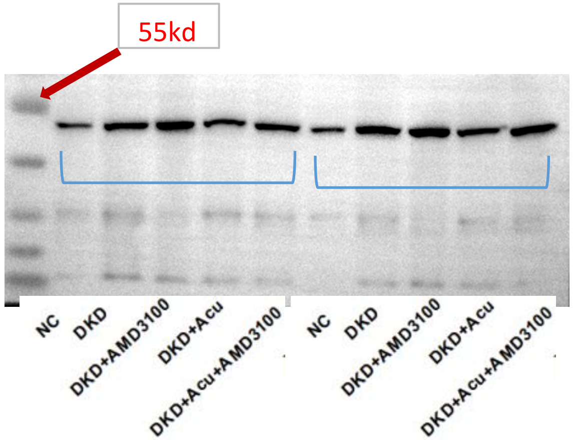

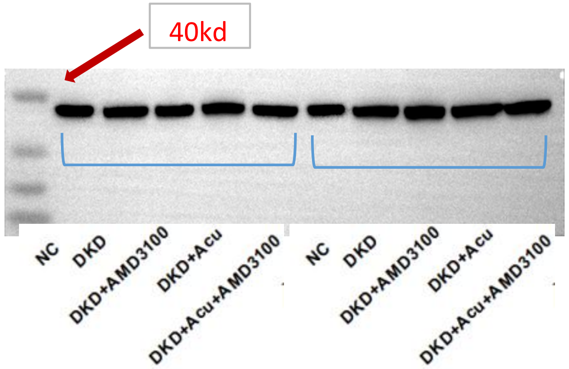

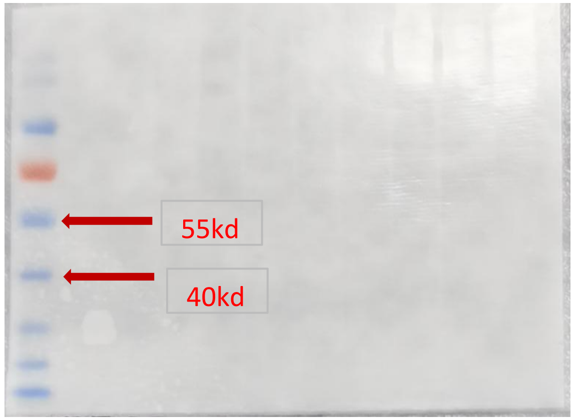

3repeat

GAPDH(37KD)

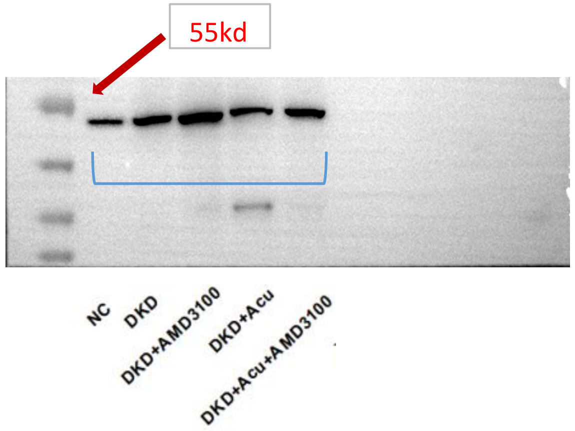

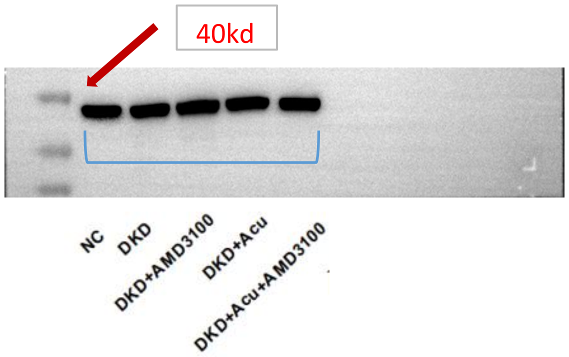

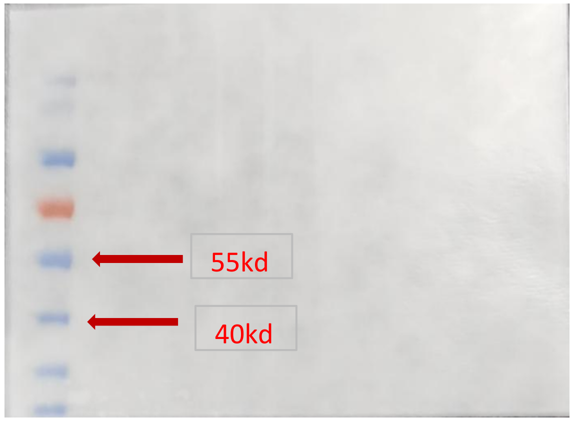

Dpp4 88kd

1~2repeat

GAPDH(37KD)

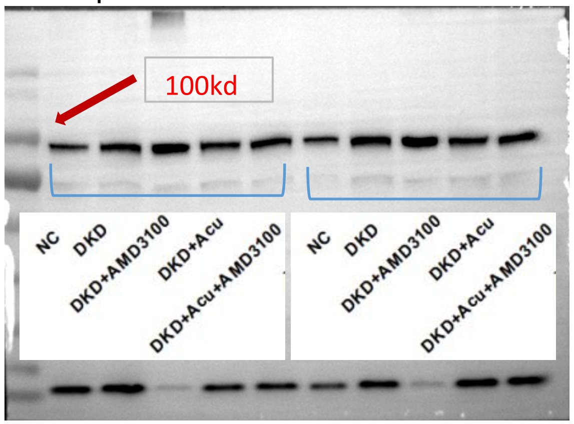

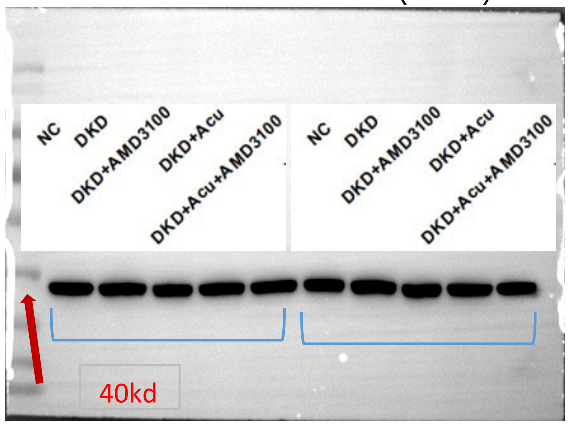

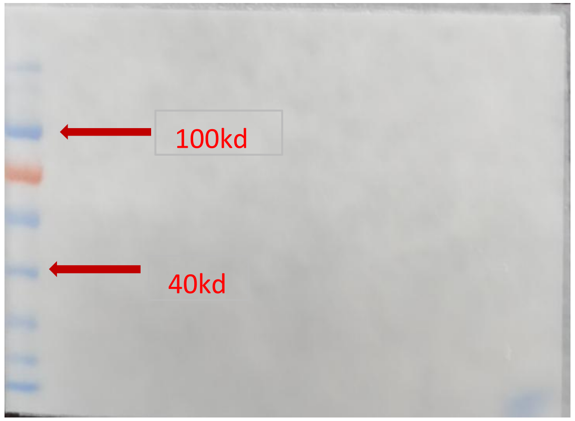

3repeat

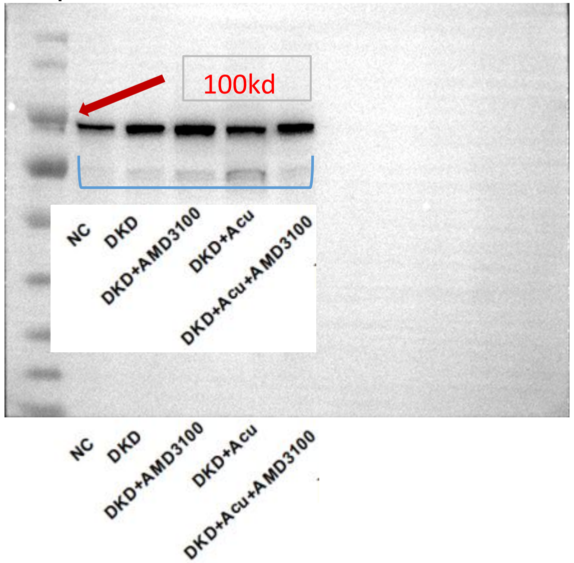

GAPDH(37KD)

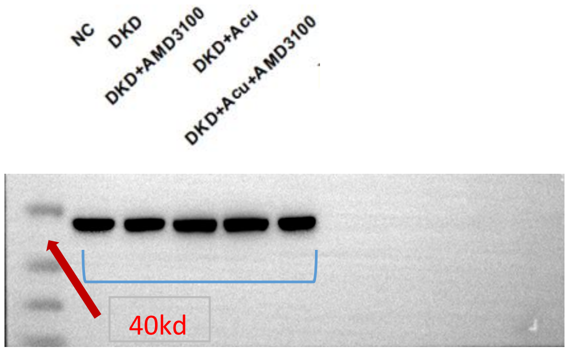

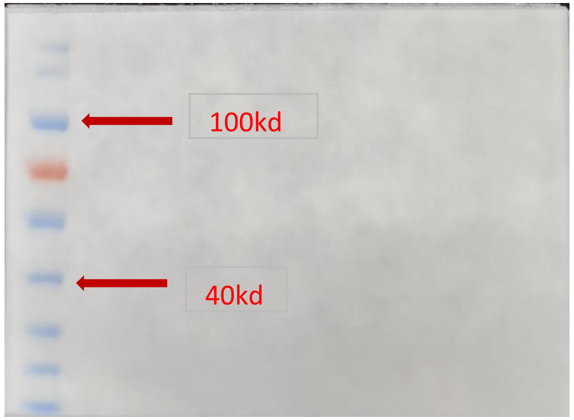

Fsp1 12kd

1~2 repeat

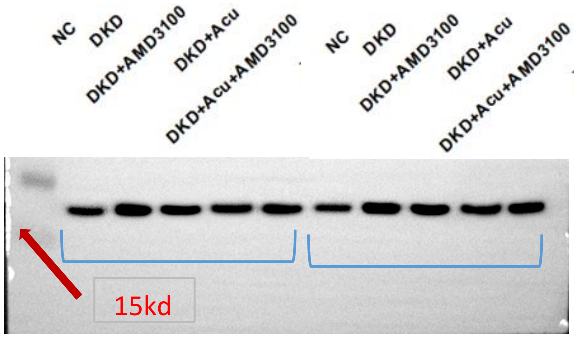

GAPDH(37KD)

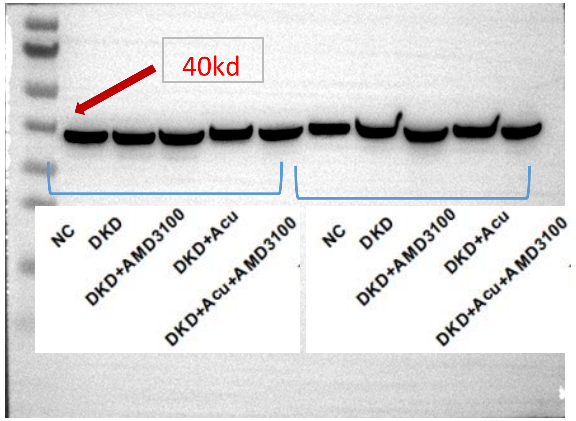

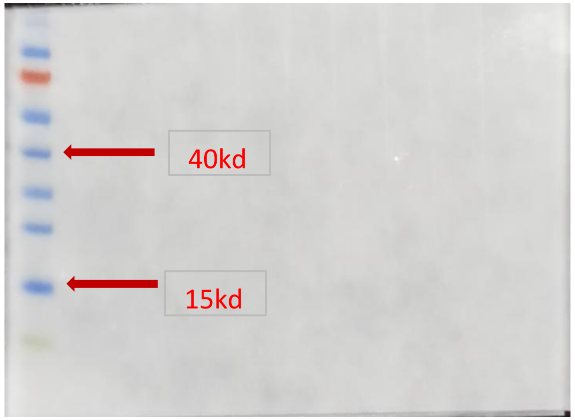

repeat3

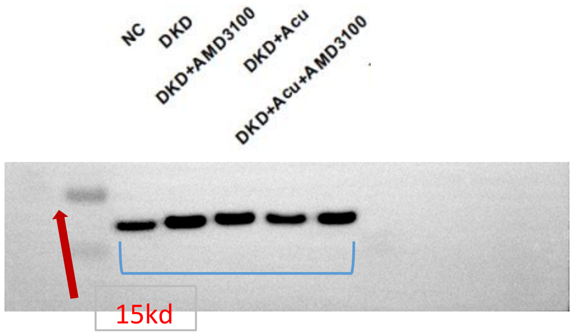

GAPDH(37KD)

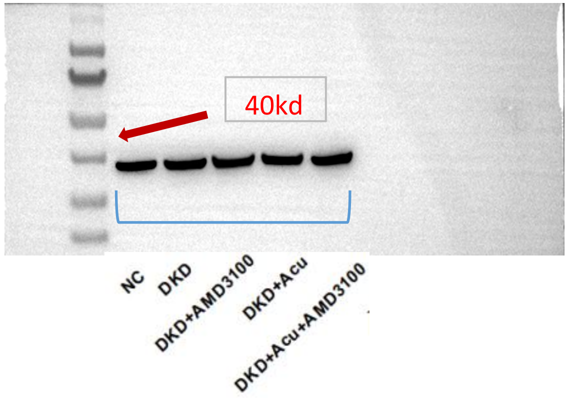

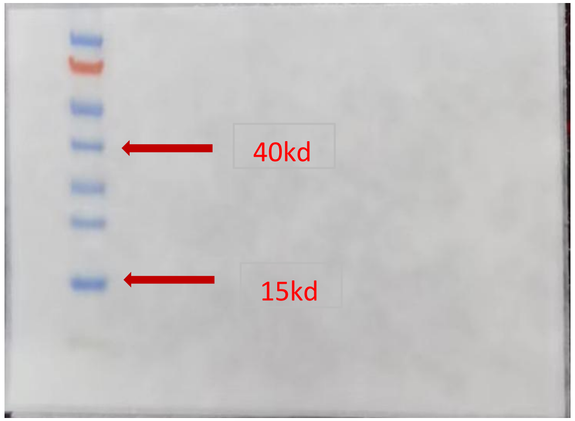

Nephrin 135kd

1~2 repeat

GAPDH(37KD)

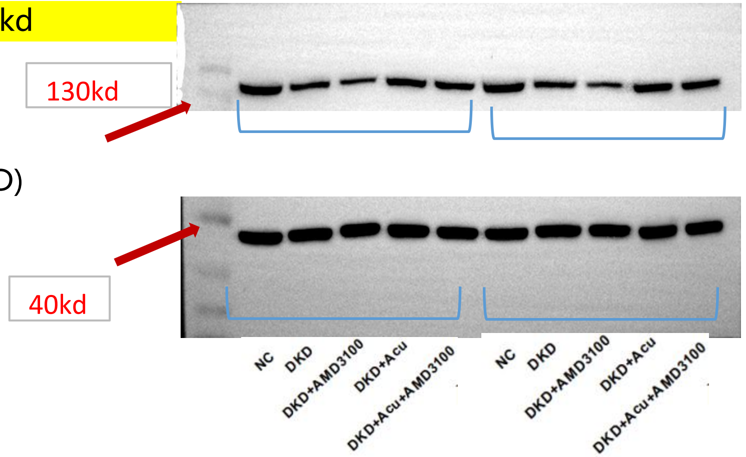

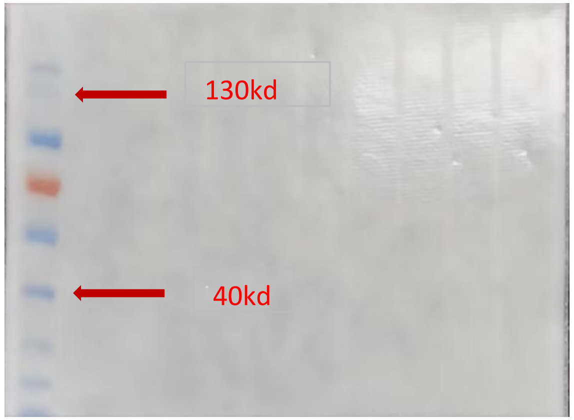

3repeat

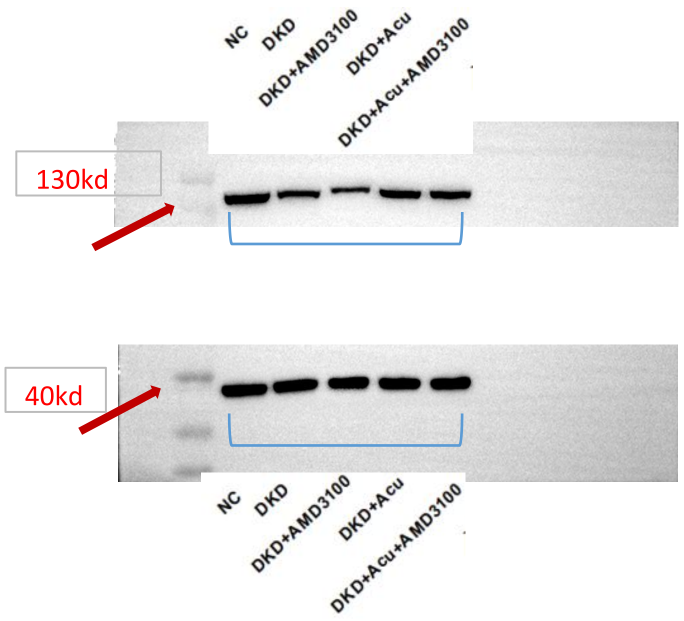

GAPDH(37KD)

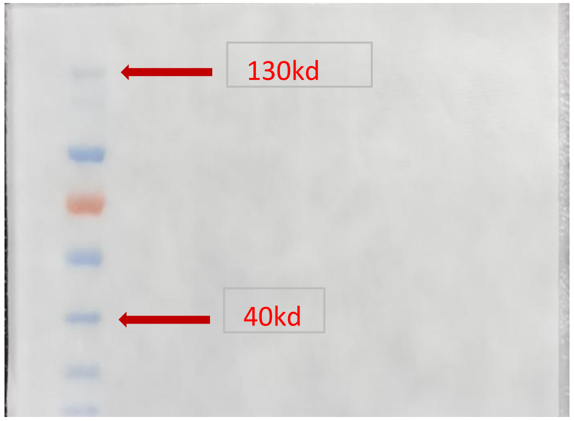

Podocin 42kd

1~2 repeat

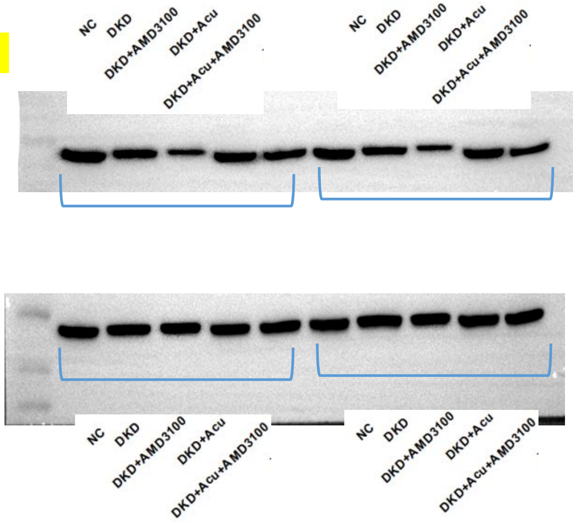

GAPDH(37KD)

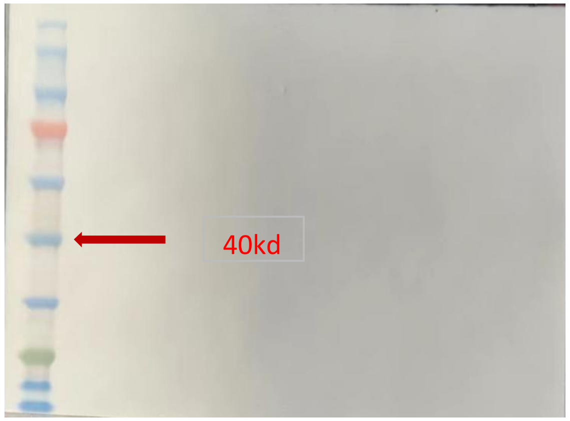

3 repeat

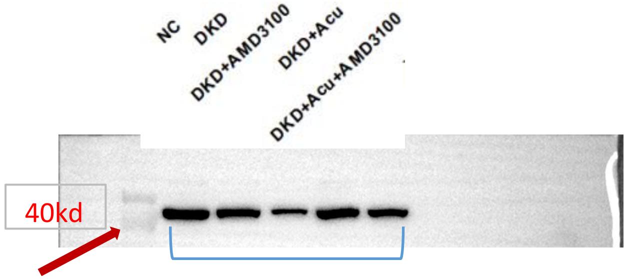

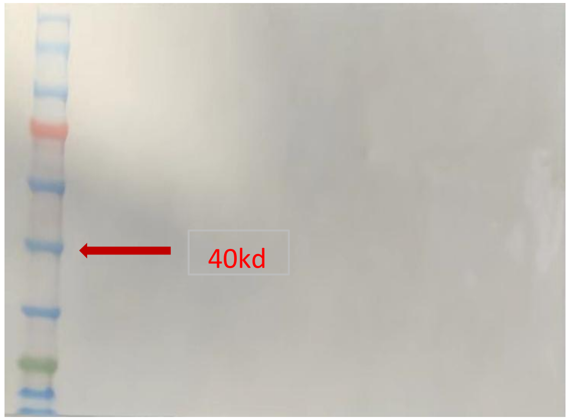

GAPDH(37KD)

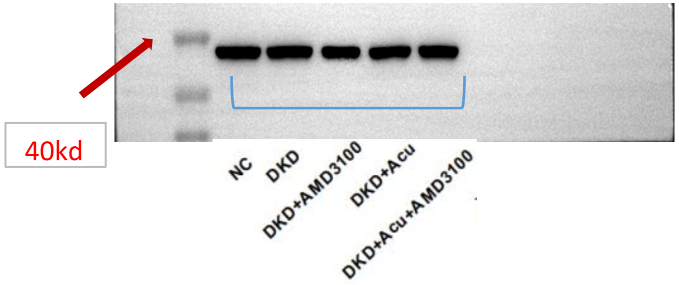

p-Smad3 50kd

1~2 repeat

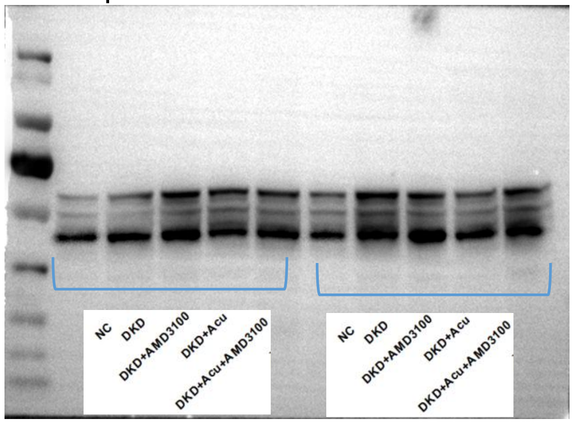

GAPDH(37KD)

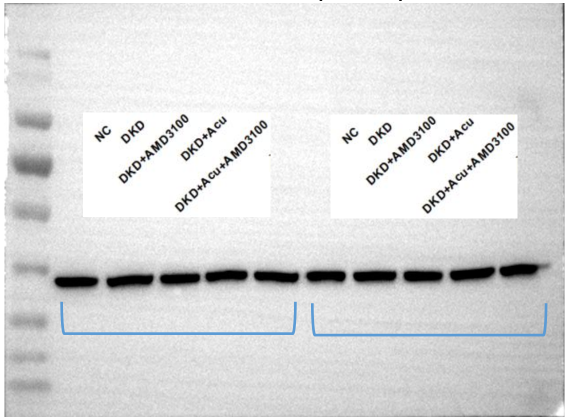

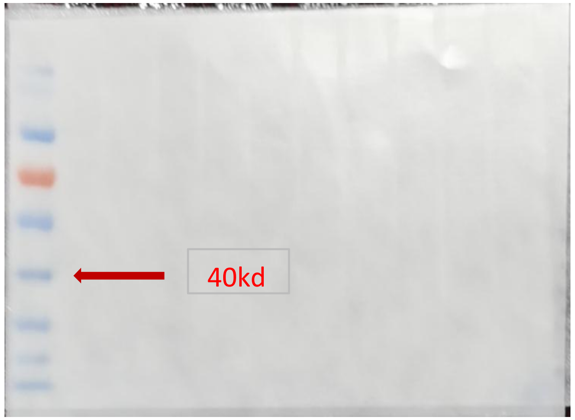

3 repeat

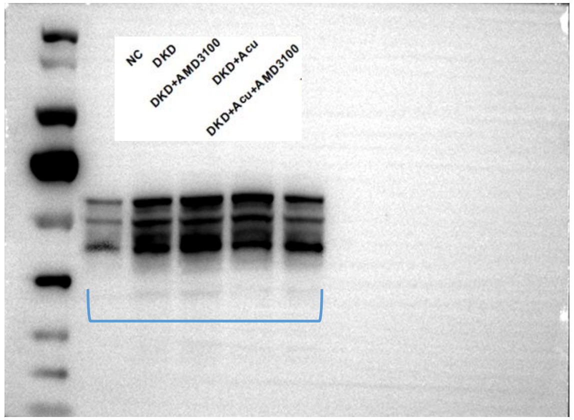

GAPDH(37KD)

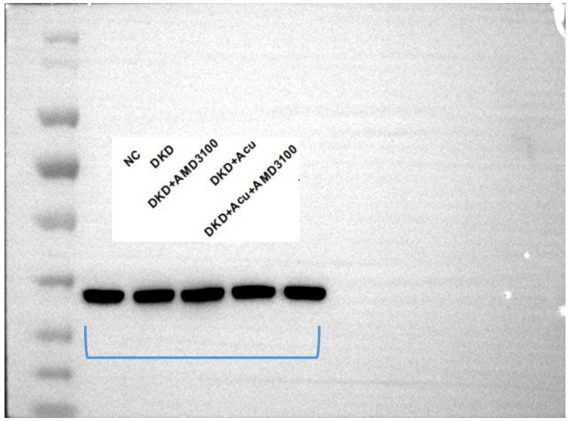

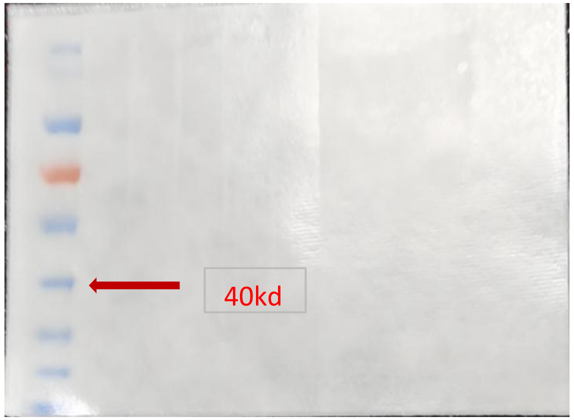

SDF-1α12kd

1~2 repeat

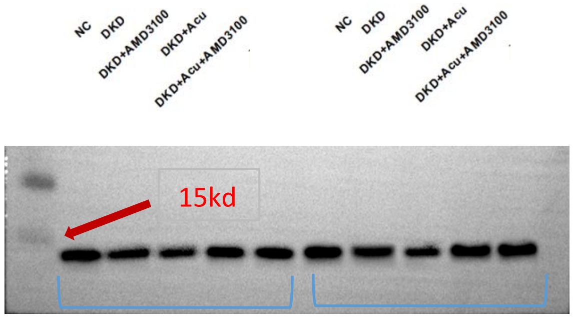

GAPDH(37KD)

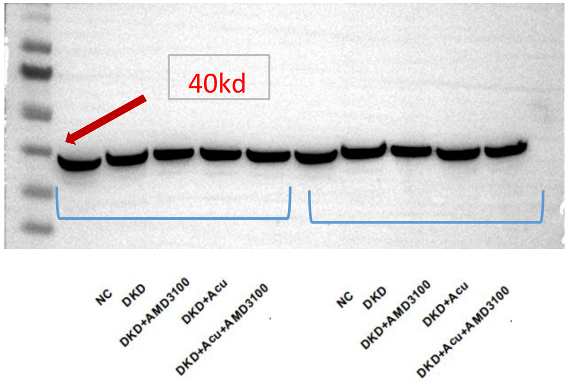

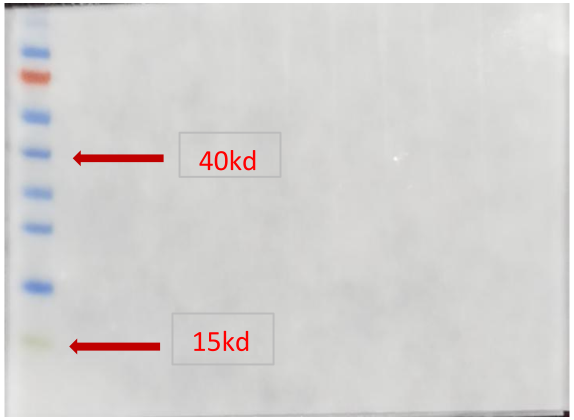

3 repeat

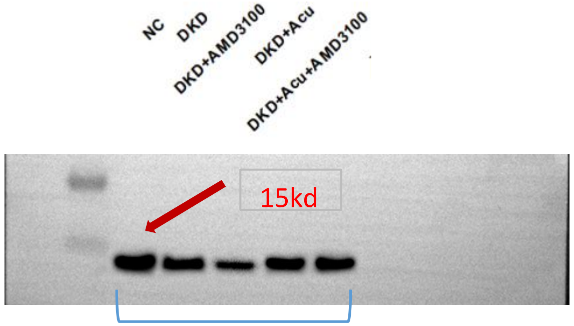

GAPDH(37KD)

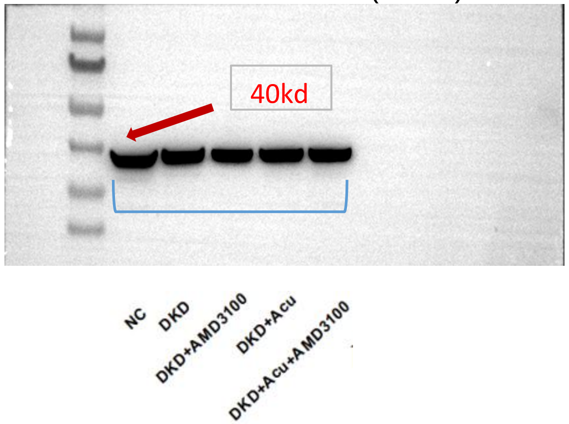

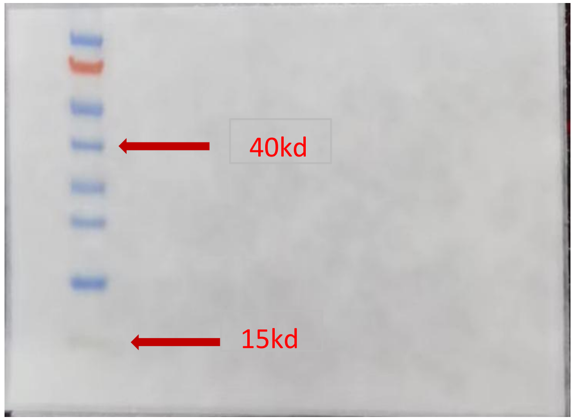

Smad3 50kd

1~2repeat

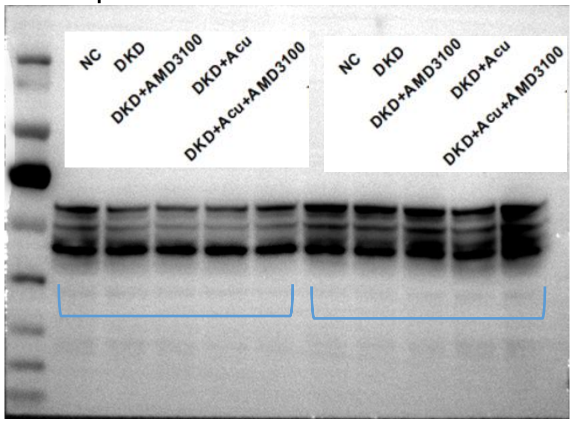

GAPDH(37KD)

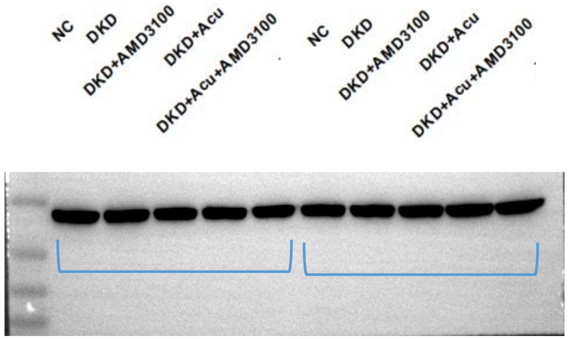

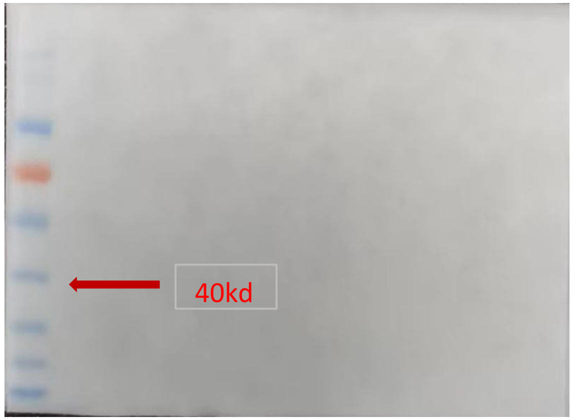

3 repeat

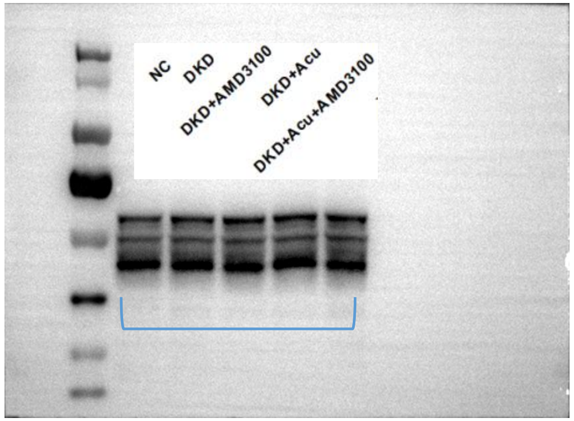

GAPDH(37KD)

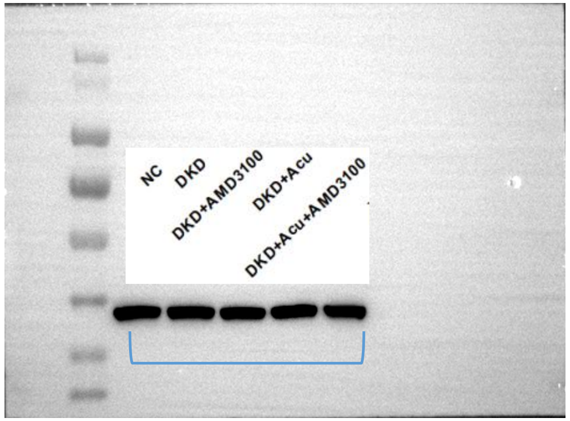

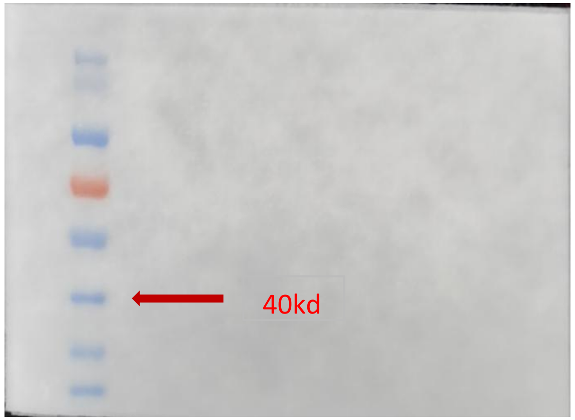

TGF-β1 14kd

1~2 repeat

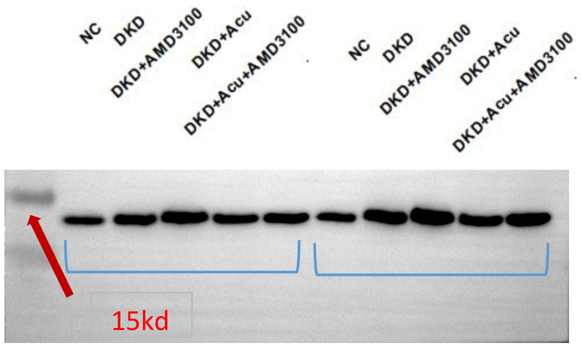

GAPDH(37KD)

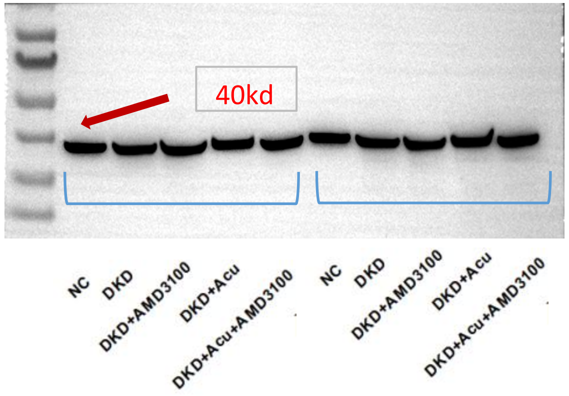

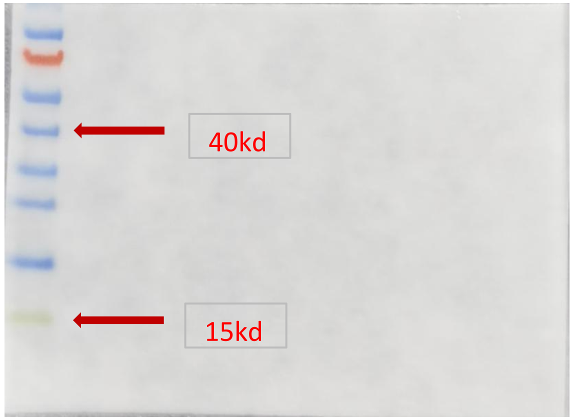

3 repeat

GAPDH(37KD)

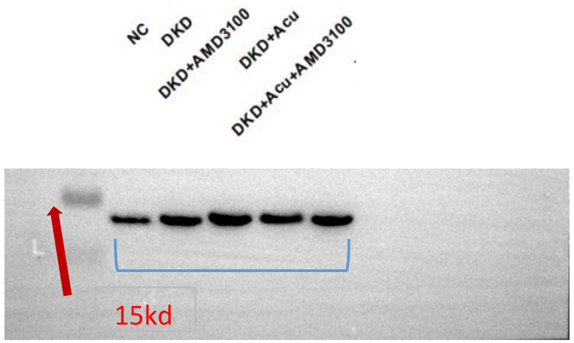

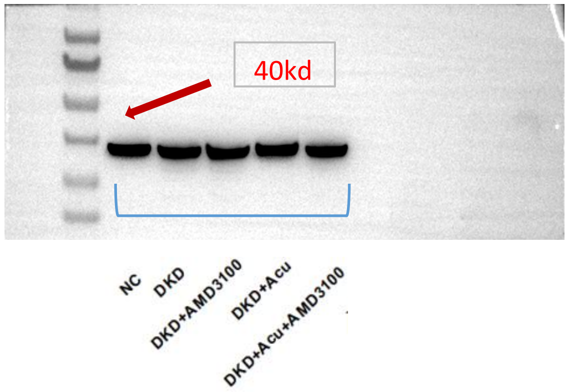

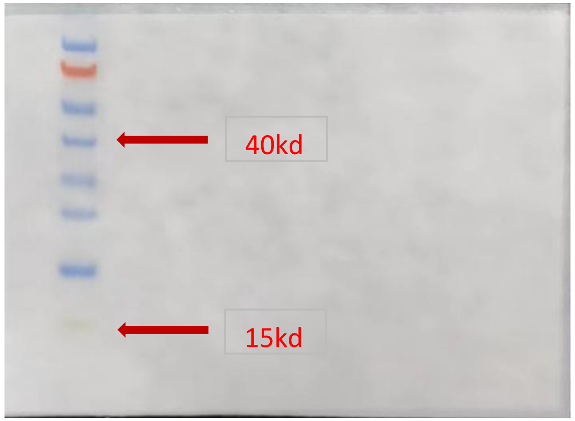

α-SMA 42kd

1~2 repeat

GAPDH(37KD)

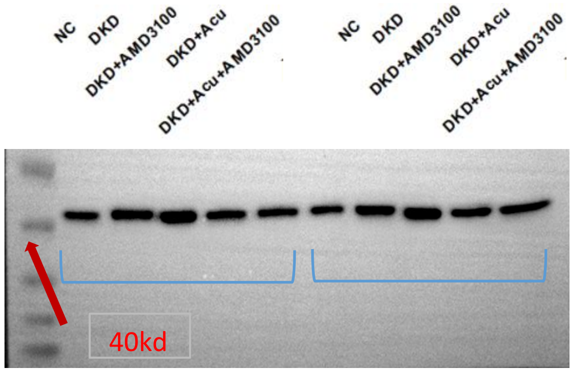

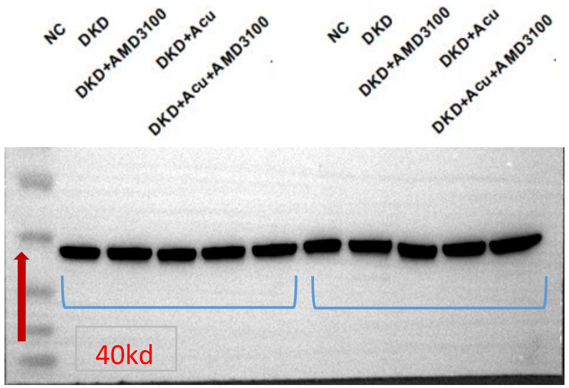

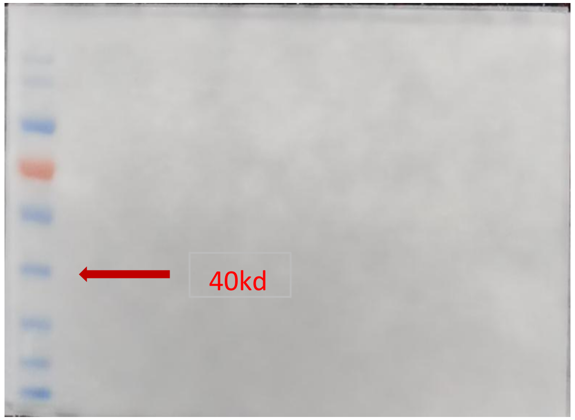

3 repeat

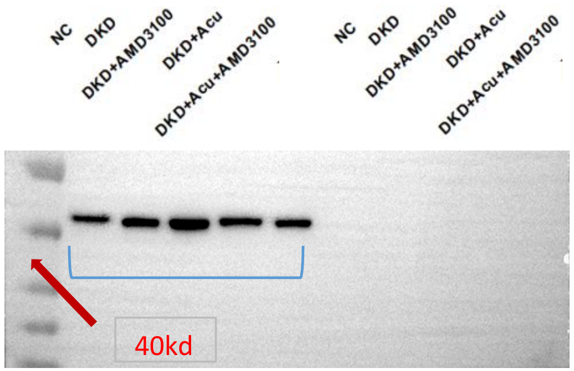

GAPDH(37KD)

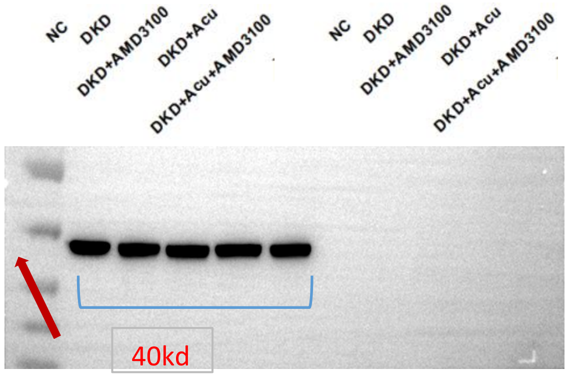

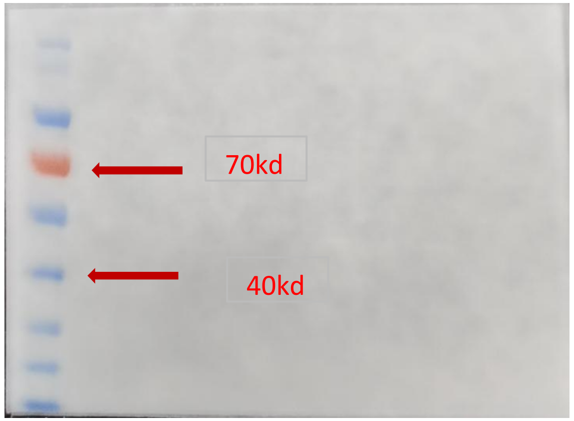

Supplement: Supporting Information 4 — Western blot. [file 2379872.f4.pdf]
