# Supplementary material for: Transcriptomics and Proteomics Reveal That TLPW Acupuncture Ameliorates Proteinuria in Diabetic Kidney Disease Model Rats by Suppressing Epithelial-to-Mesenchymal Transition via the DPP4/SDF-1α/TGF-β/Smad Signalling Axis
Source: J Diabetes Res. 2025 Oct 1;2025:2379872. doi: 10.1155/jdr/2379872 (PMC12507495; doi:10.1155/jdr/2379872)
Supplement: Supporting Information 2 — Periodic acid–Schiff (PAS) staining. [file 2379872.f2.doc]

Control group

DKD group

DKD+ACU
